# Supplementary material for: Ecophysiological adaptations shape distributions of closely related trees along a climatic moisture gradient
Source: Nat Commun. 2023 Nov 7;14:7173. doi: 10.1038/s41467-023-42352-w (PMC10630429; doi:10.1038/s41467-023-42352-w)
Supplement: Supplementary file 1 — Supplementary Infromation [file 41467_2023_42352_MOESM1_ESM.pdf]

Online Supplement to:

Ecophysiological adaptations shape distributions of closely related trees along a climatic moisture gradient

Duncan D. Smith[1,2,3], Mark A. Adams[2], Amanda M. Salvi[1], Christopher P. Krieg[1], Cécile Ané[1,4], Katherine A. McCulloh[1], Thomas J. Givnish[1]

[1]Department of Botany, University of Wisconsin-Madison, Madison, WI 53706, USA

[2]Faculty of Science, Engineering, & Technology, Swinburne University of Technology, Hawthorn, Victoria 3122, Australia

[3]School of Ecosystem and Forest Sciences, University of Melbourne, Creswick, Victoria 3363 Australia

[4]Department of Statistics, University of Wisconsin-Madison, Madison, WI 53706, USA

Corresponding authors: Duncan D. Smith (ddsmith3@wisc.edu)

Thomas J. Givnish (givnish@wisc.edu)

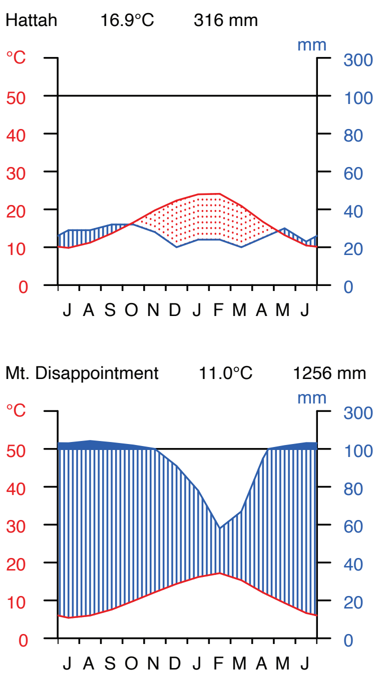

Figure S1. Climate diagrams for the four study sites. Each diagram plots mean monthly temperature and rainfall against time, at a scale of 10°C = 20 mm; monthly precipitation >100 mm is plotted at 10% scale. Mean annual temperature and precipitation are shown in the title lines; monthly mean data for the reference period (1970-2000) are given in Table S3. Stripes indicate relatively moist periods; stippling, relatively dry periods. Note small monthly dip in precipitation in the southern hemisphere summer. Source data are provided as a Source Data file.

Figure S2. Phylogenetically structured fit of all traits to regressions based on site and species P/E_p, and partial effects due to site P/E_p alone and species P/E_p alone. For each trait, the first two graphs show the partial effects of site and species P/E_p, while the contour map shows the trait as a function of both parameters. On the left, species are color-coded from driest (red) to moistest (purple) native habitats; in the middle, gardens are color-coded from driest (tan) to moistest (blue). See nine following pages. Solid curves indicate significant relationships ($p<0.05$) with the variable on the x-axis; non-significant fits are shown as dashed curves. Source data including trait definitions are provided as a Source Data file.

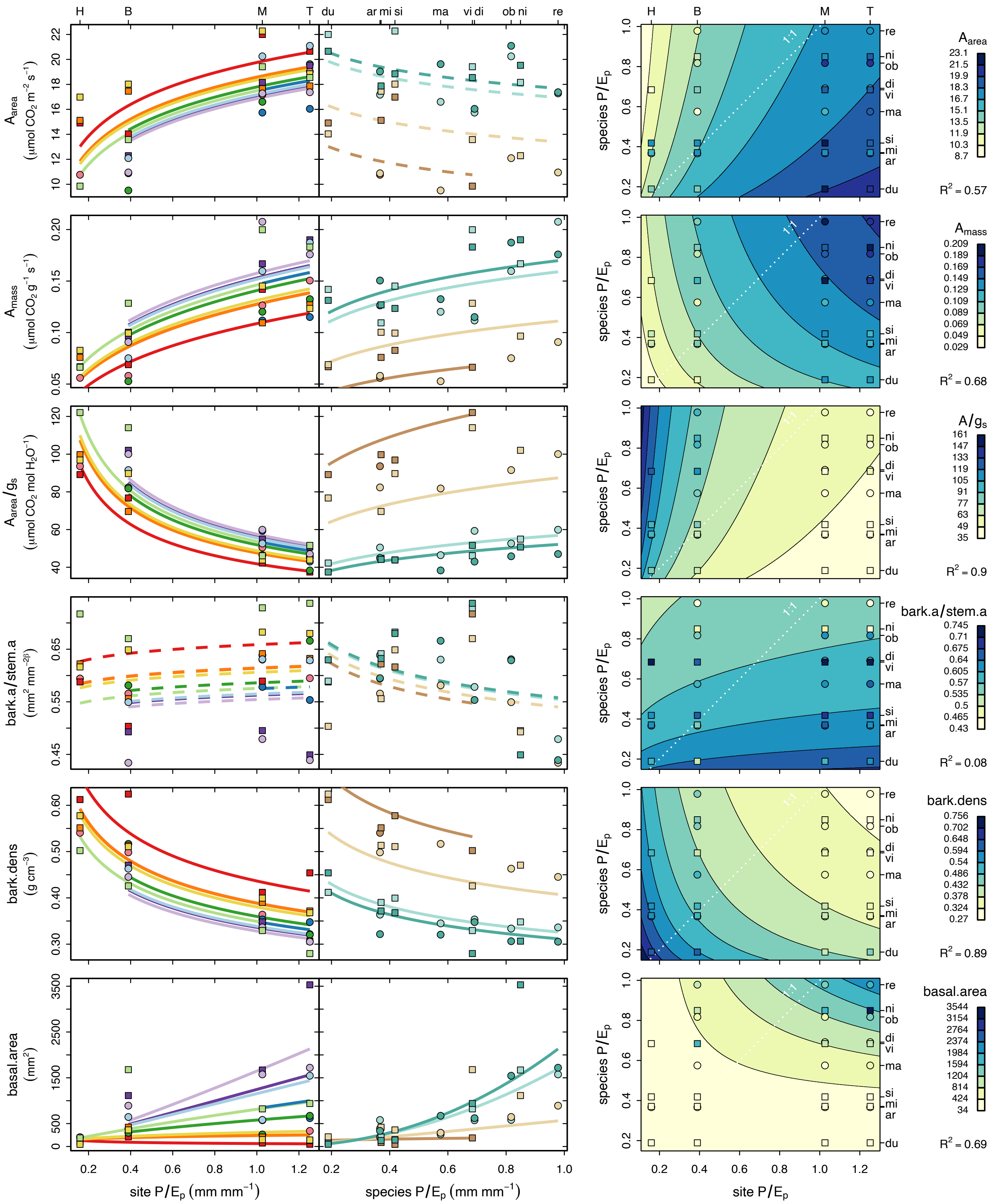

H B M T
du ar mi si ma vi di ob ni re
A_{area} (μmol CO_2 m^{-2} s^{-1})
A_{mass} (μmol CO_2 g^{-1} s^{-1})
A_{area}/g_s (μmol CO_2 mol H_2O^{-1})
bark.a/stem.a (mm^2 $mm^{-2\beta}$)
bark.dens (g cm^{-3})
basal.area (mm^2)
site P/E_p (mm mm^{-1})
species P/E_p (mm mm^{-1})
species P/E_p
site P/E_p
1:1
A_{area}
$R^2 = 0.57$
A_{mass}
$R^2 = 0.68$
A/g_s
$R^2 = 0.9$
bark.a/stem.a
$R^2 = 0.08$
bark.dens
$R^2 = 0.89$
basal.area
$R^2 = 0.69$

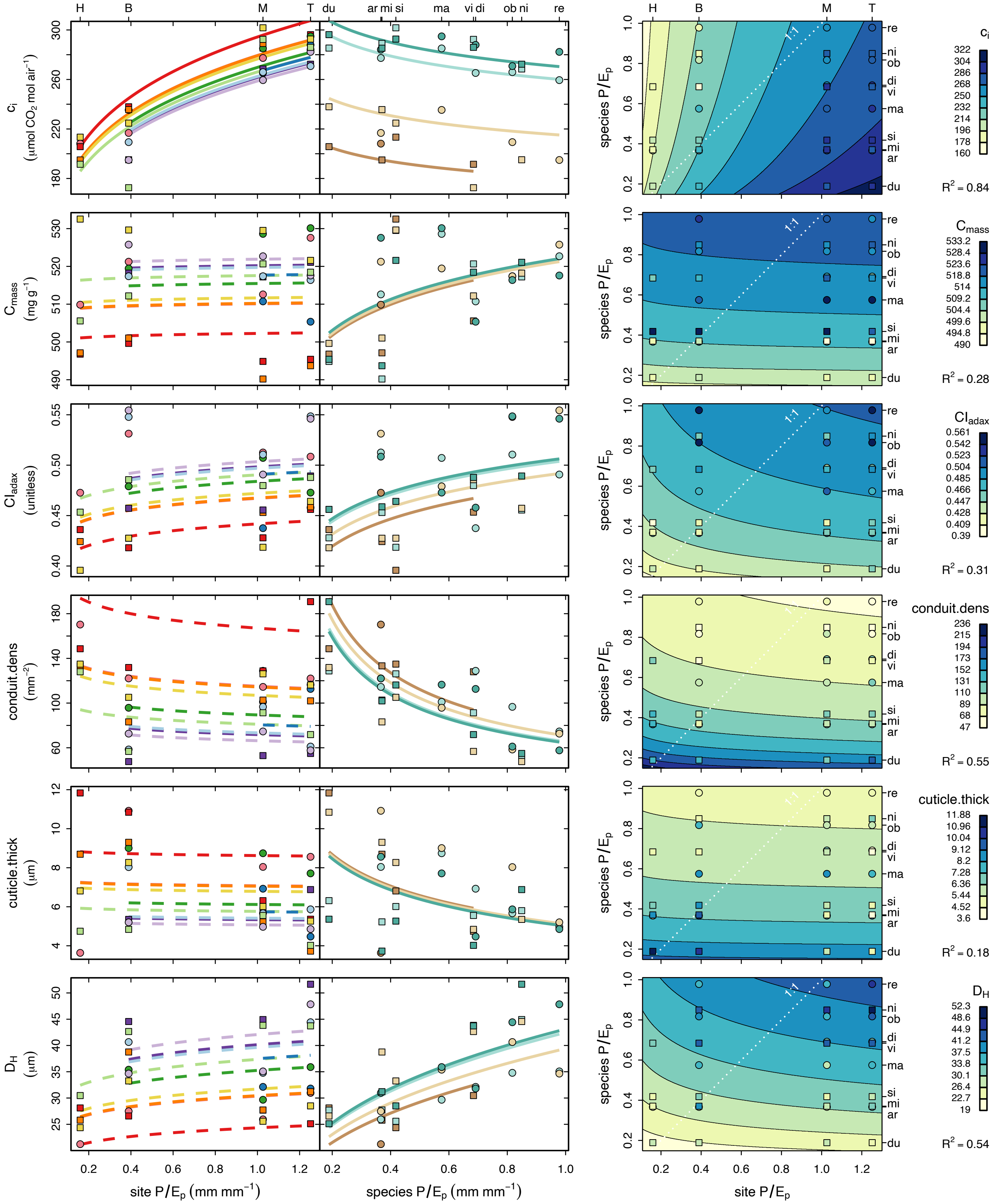

H B M T
du ar mi si ma vi di ob ni re
c_i (μmol CO_2 mol air^{-1})
C_{mass} (mg g^{-1})
CI_{adax} (unitless)
conduit.dens (mm^{-2})
cuticle.thick (μm)
D_H (μm)
site P/E_p (mm mm^{-1})
species P/E_p (mm mm^{-1})
species P/E_p
site P/E_p
1:1
R^2 = 0.84
R^2 = 0.28
R^2 = 0.31
R^2 = 0.55
R^2 = 0.18
R^2 = 0.54

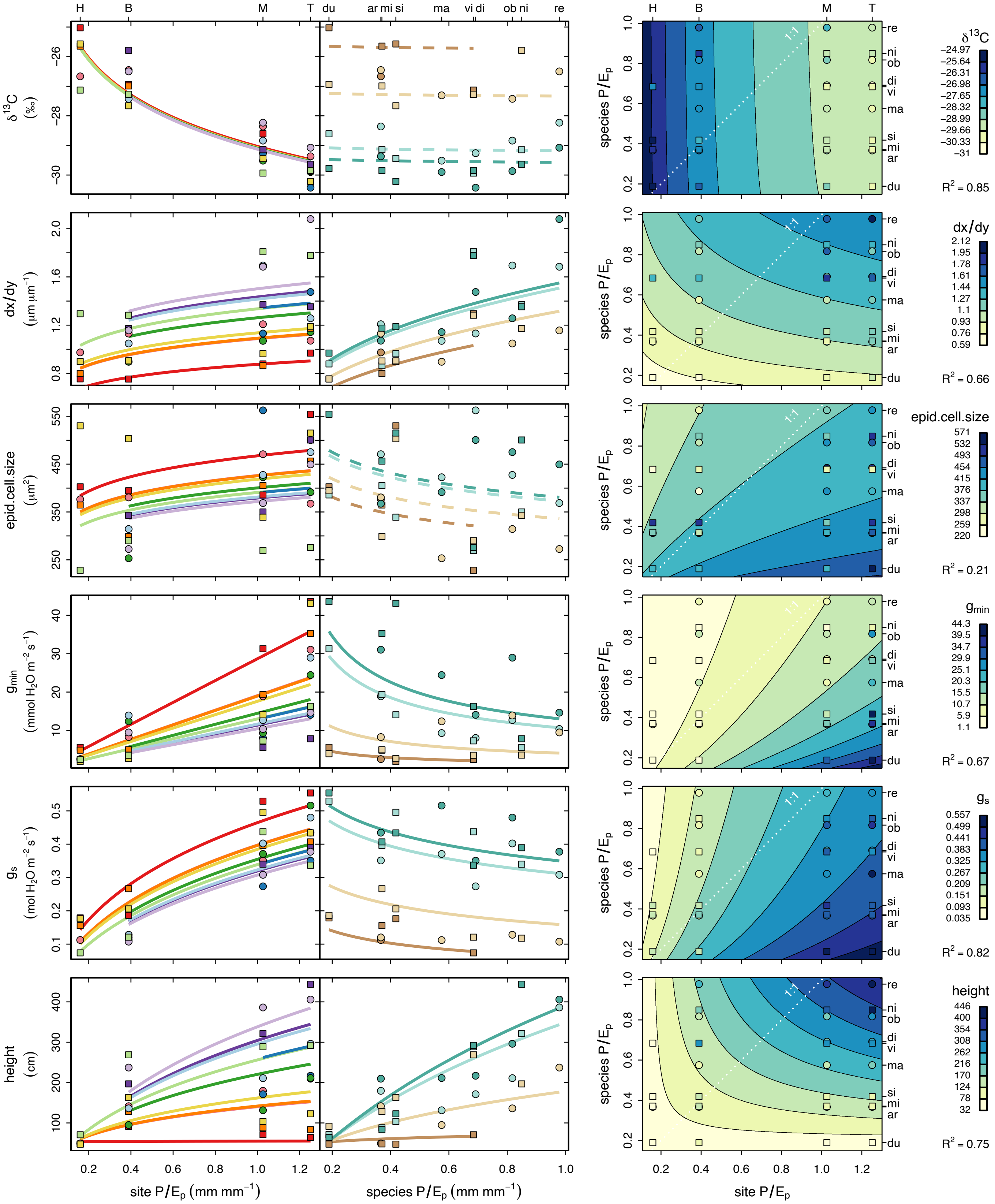

H
B
M
T
du
ar mi si
ma
vi di
ob ni
re
δ13C (‰)
dx/dy (μm μm−1)
epid.cell.size (μm2)
gmin (mmol H2O m−2 s−1)
gs (mol H2O m−2 s−1)
height (cm)
site P/Ep (mm mm−1)
species P/Ep (mm mm−1)
species P/Ep
site P/Ep
1:1
δ13C
R2 = 0.85
dx/dy
R2 = 0.66
epid.cell.size
R2 = 0.21
gmin
R2 = 0.67
gs
R2 = 0.82
height
R2 = 0.75

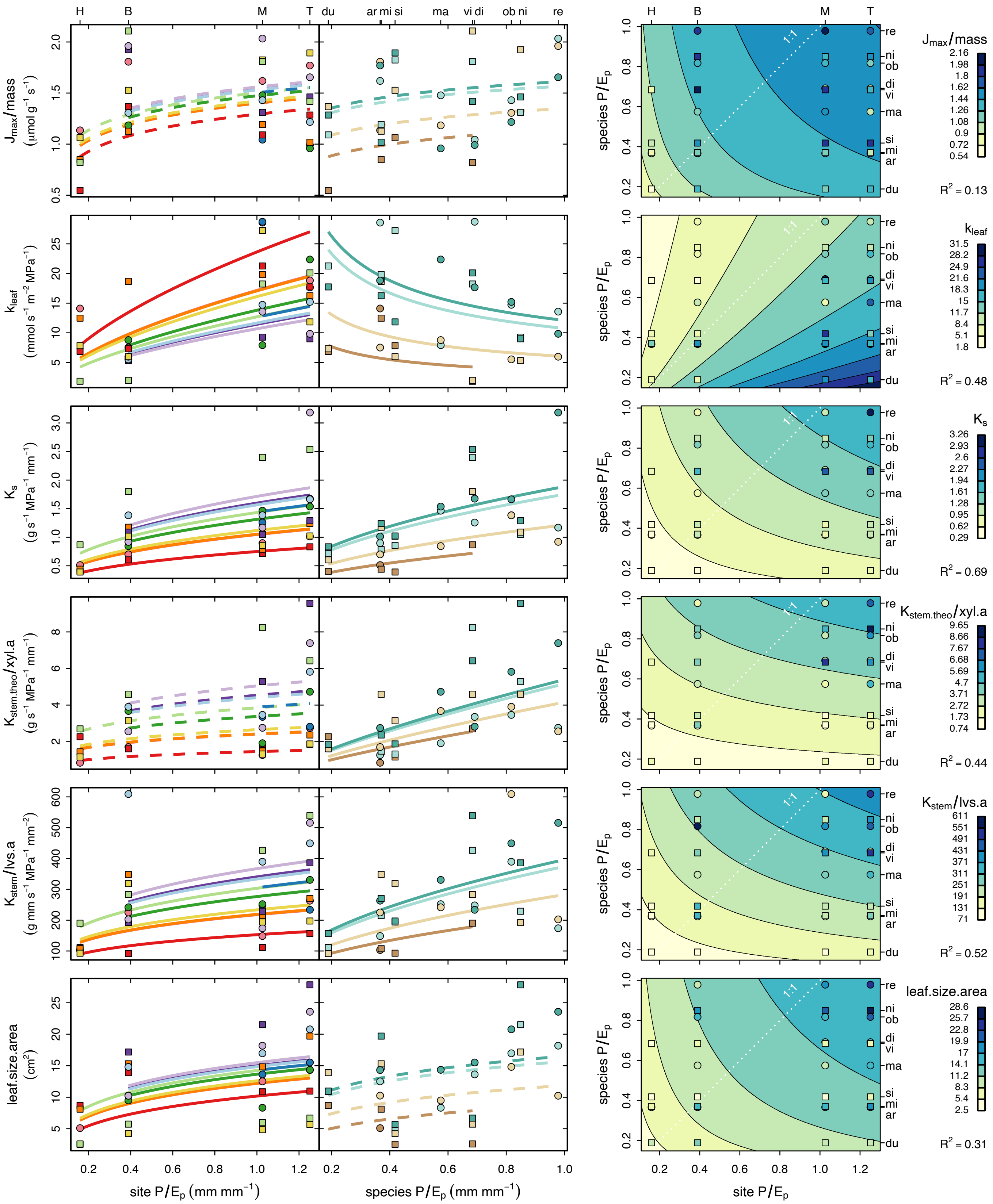

H
B
M
T
du
ar mi si
ma
vi di
ob ni
re
J_{max}/mass (μmol g^{-1} s^{-1})
k_{leaf} (mmol s^{-1} m^{-2} MPa^{-1})
K_s (g s^{-1} MPa^{-1} mm^{-1})
$K_{stem.theo}$/xyl.a (g s^{-1} MPa^{-1} mm^{-1})
K_{stem}/lvs.a (g mm s^{-1} MPa^{-1} mm^{-2})
leaf.size.area (cm^2)
site P/E_p (mm mm^{-1})
species P/E_p (mm mm^{-1})
species P/E_p
site P/E_p
1:1
J_{max}/mass
R^2 = 0.13
k_{leaf}
R^2 = 0.48
K_s
R^2 = 0.69
$K_{stem.theo}$/xyl.a
R^2 = 0.44
K_{stem}/lvs.a
R^2 = 0.52
leaf.size.area
R^2 = 0.31

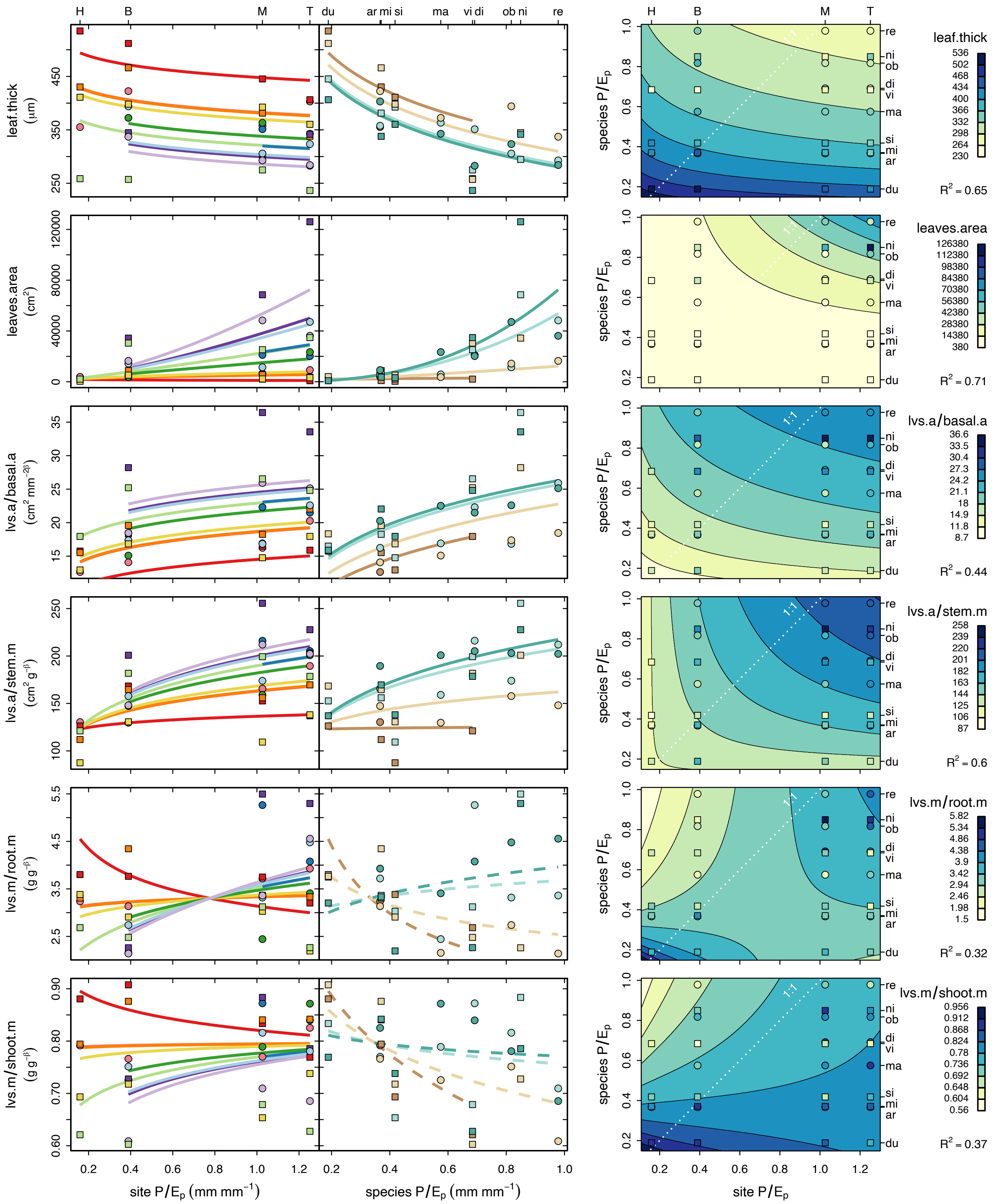
H
B
M
T
du
ar mi si
ma
vi di
ob ni
re
leaf.thick
(μm)
leaves.area
(cm²)
lvs.a/basal.a
(cm² mm⁻²ᵝ)
lvs.a/stem.m
(cm² g⁻ᵝ)
lvs.m/root.m
(g g⁻ᵝ)
lvs.m/shoot.m
(g g⁻ᵝ)
site P/Ep (mm mm⁻¹)
species P/Ep (mm mm⁻¹)
species P/Ep
site P/Ep
1:1
R² = 0.65
R² = 0.71
R² = 0.44
R² = 0.6
R² = 0.32
R² = 0.37

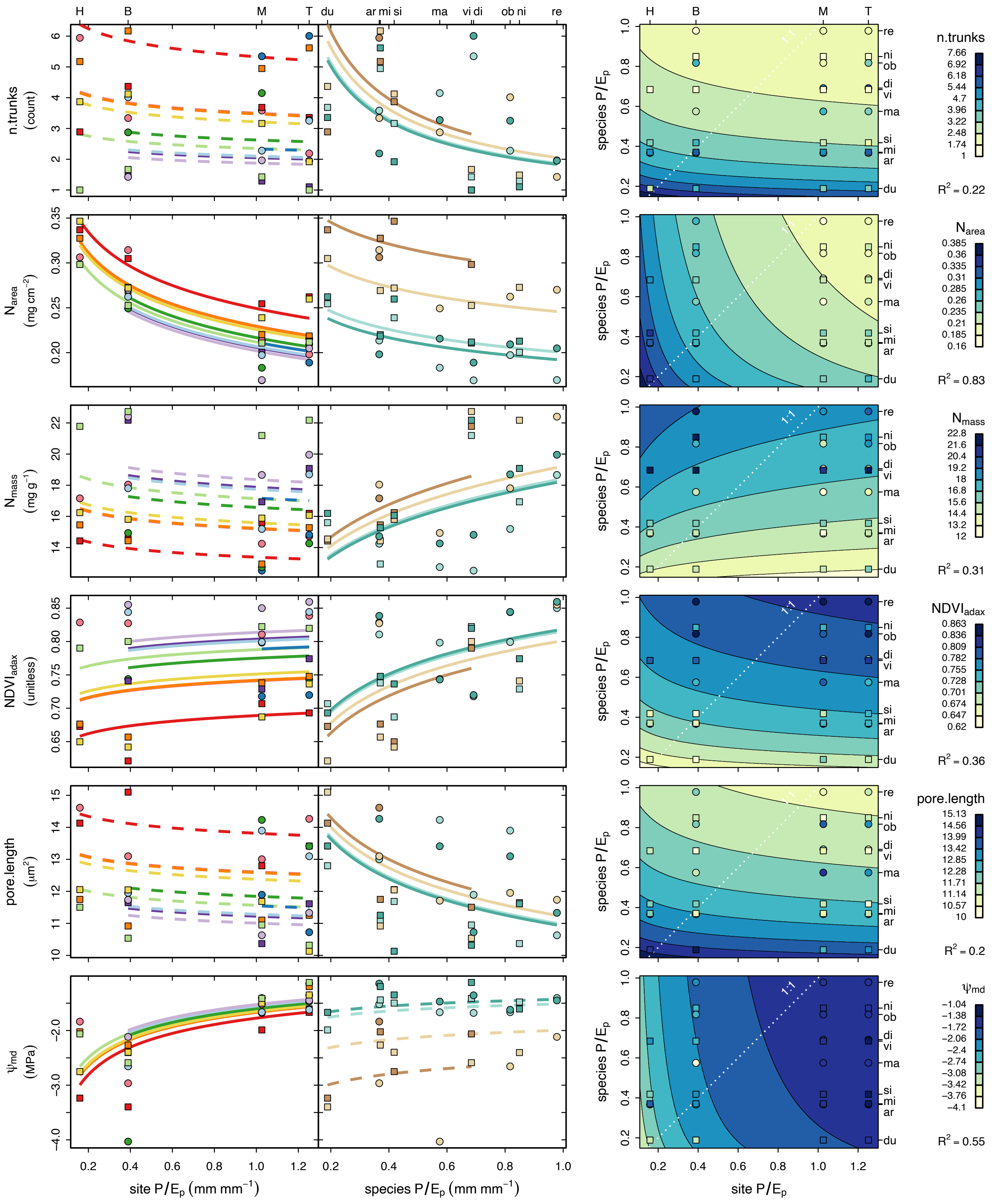

H
B
M
T
du
ar mi si
ma
vi di
ob ni
re
n.trunks (count)
N_{area} (mg cm^{-2})
N_{mass} (mg g^{-1})
$NDVI_{adax}$ (unitless)
pore.length (μm^2)
ψ_{md} (MPa)
site P/E_p (mm mm^{-1})
species P/E_p (mm mm^{-1})
species P/E_p
site P/E_p
1:1
n.trunks
$R^2 = 0.22$
N_{area}
$R^2 = 0.83$
N_{mass}
$R^2 = 0.31$
$NDVI_{adax}$
$R^2 = 0.36$
pore.length
$R^2 = 0.2$
ψ_{md}
$R^2 = 0.55$

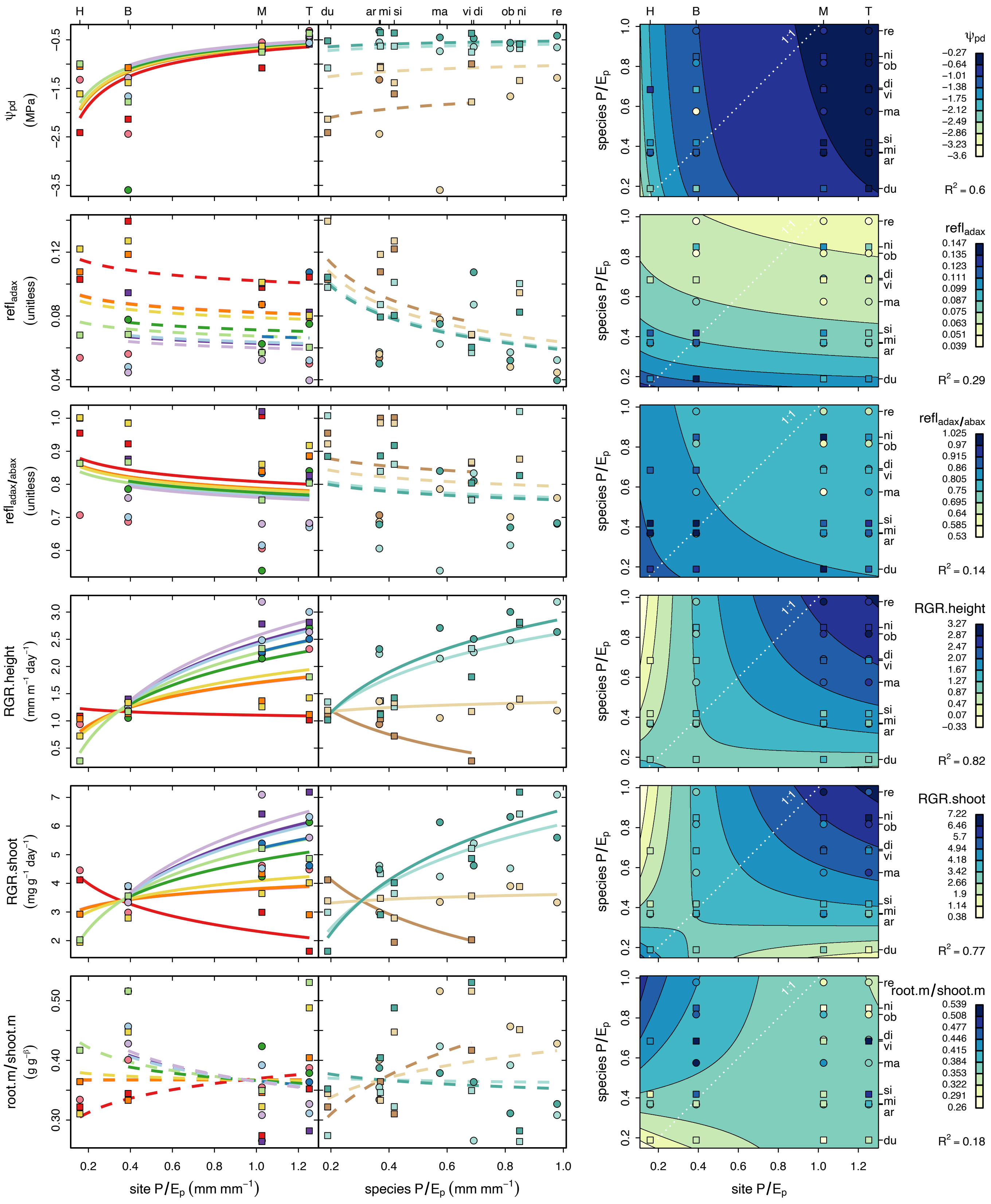

H
B
M
T
du
ar mi si
ma
vi di
ob ni
re
ψpd (MPa)
refladax (unitless)
refladax/abax (unitless)
RGR.height (mm m^-1 day^-1)
RGR.shoot (mg g^-1 day^-1)
root.m/shoot.m (g g^-β)
site P/Ep (mm mm^-1)
species P/Ep (mm mm^-1)
species P/Ep
site P/Ep
1:1
ψpd
R^2 = 0.6
refladax
R^2 = 0.29
refladax/abax
R^2 = 0.14
RGR.height
R^2 = 0.82
RGR.shoot
R^2 = 0.77
root.m/shoot.m
R^2 = 0.18

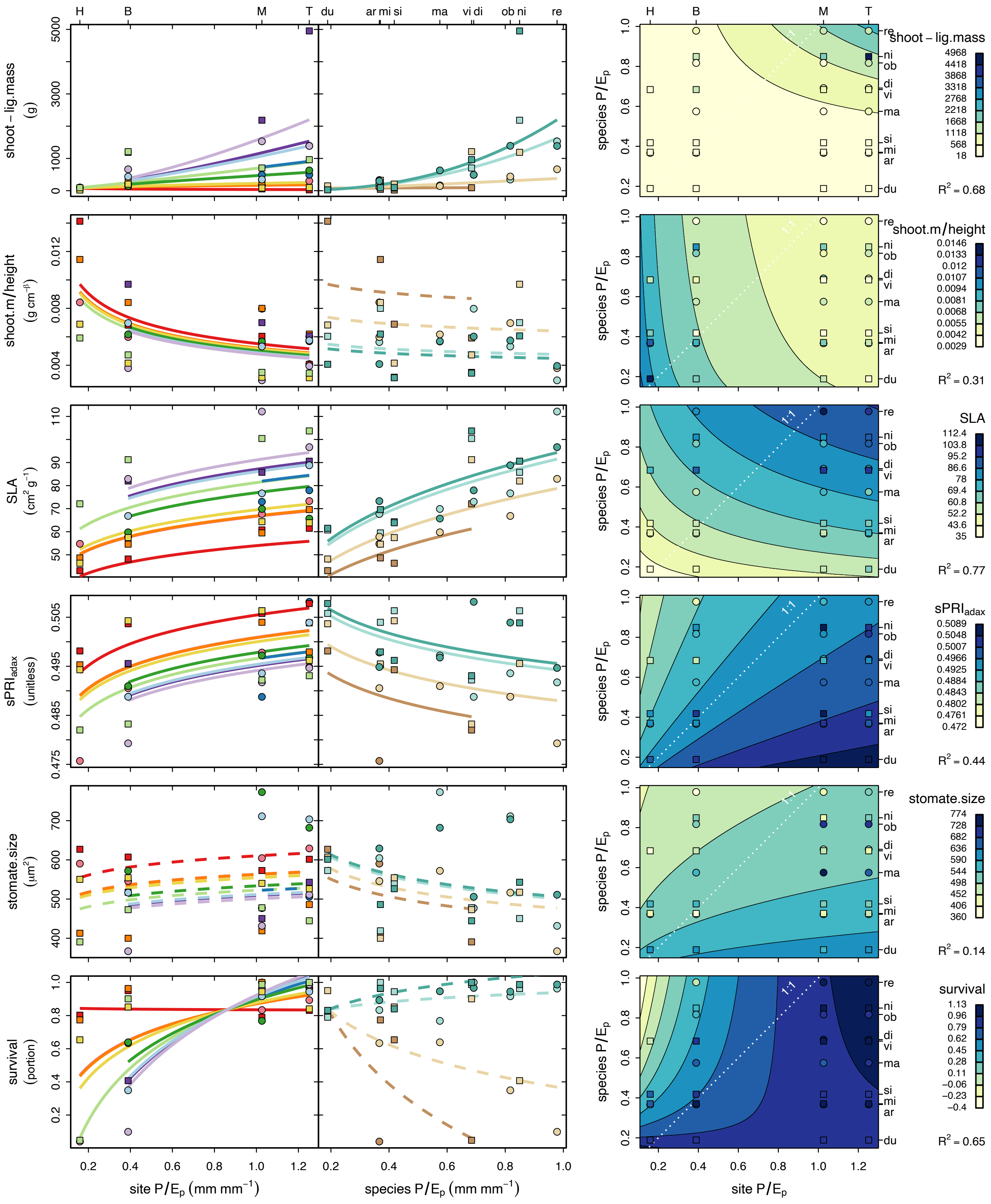
shoot – lig.mass (g)
shoot.m/height (g cm⁻³)
SLA (cm² g⁻¹)
sPRIadax (unitless)
stomate.size (µm²)
survival (portion)
H
B
M
T
du
ar mi si
ma
vi di
ob ni
re
site P/Ep (mm mm⁻¹)
species P/Ep (mm mm⁻¹)
species P/Ep
site P/Ep
1:1
shoot – lig.mass
R² = 0.68
shoot.m/height
R² = 0.31
SLA
R² = 0.77
sPRIadax
R² = 0.44
stomate.size
R² = 0.14
survival
R² = 0.65

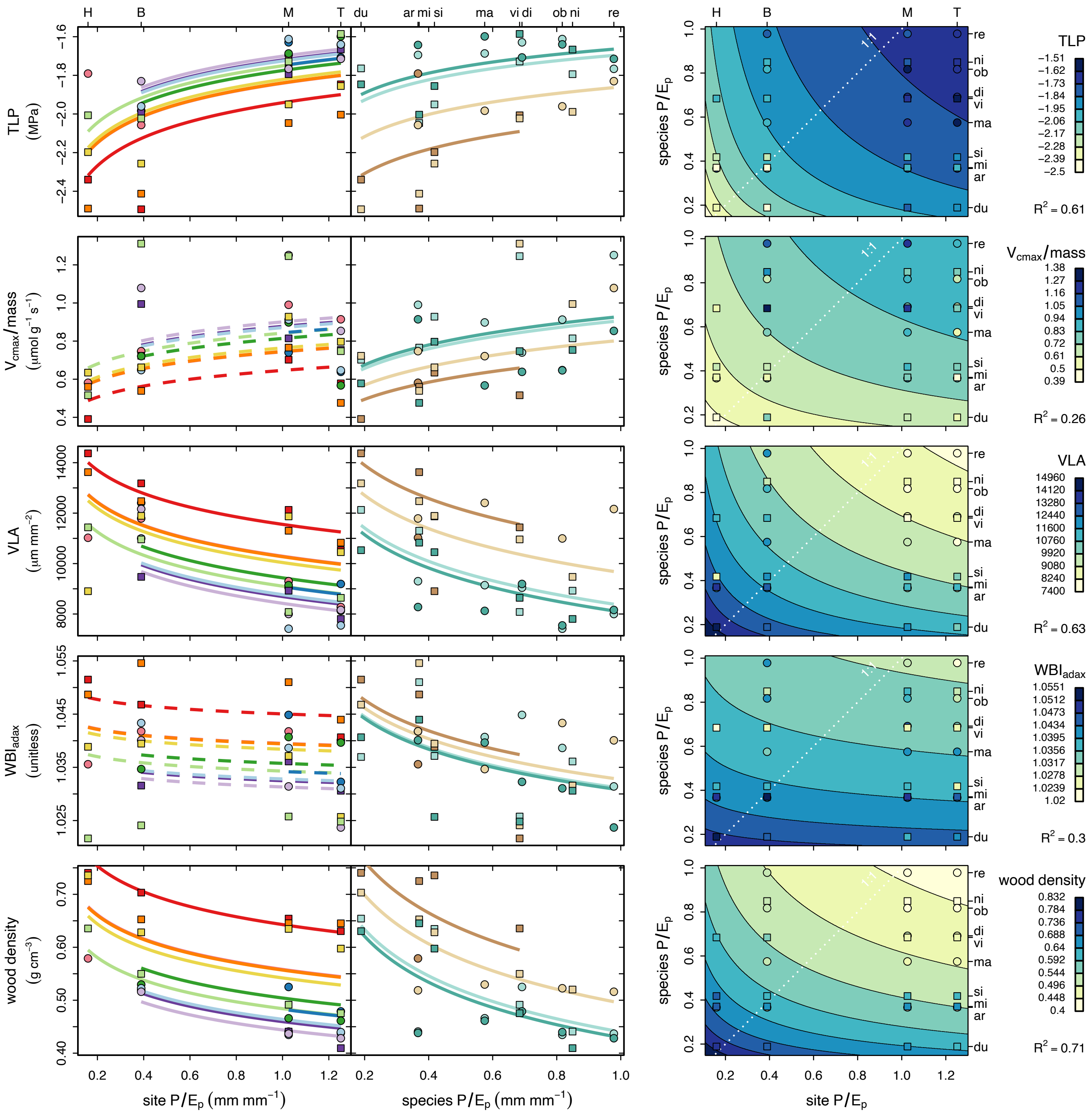

TLP (MPa)
V_{cmax}/mass (μmol g^{-1} s^{-1})
VLA (μm mm^{-2})
WBI_{adax} (unitless)
wood density (g cm^{-3})
site P/E_p (mm mm^{-1})
species P/E_p (mm mm^{-1})
species P/E_p
site P/E_p
H
B
M
T
du
ar
mi
si
ma
vi
di
ob
ni
re
1:1
TLP
R^2 = 0.61
V_{cmax}/mass
R^2 = 0.26
VLA
R^2 = 0.63
WBI_{adax}
R^2 = 0.3
wood density
R^2 = 0.71

Figure S3. Phylogenetically unstructured fit of all traits to regressions based on site and species P/E_p, and partial effects due to site P/E_p alone and species P/E_p alone. For each trait, the first two graphs show the partial effects of site and species P/E_p, while the contour map shows the trait as a function of both parameters. On the left, species are color-coded from driest (red) to moistest (purple) native habitats; in the middle, gardens are color-coded from driest (tan) to moistest (blue). Solid curves indicate significant relationships ($p<0.05$) with the variable on the x-axis; non-significant fits are shown as dashed curves. Source data are provided as a Source Data file.

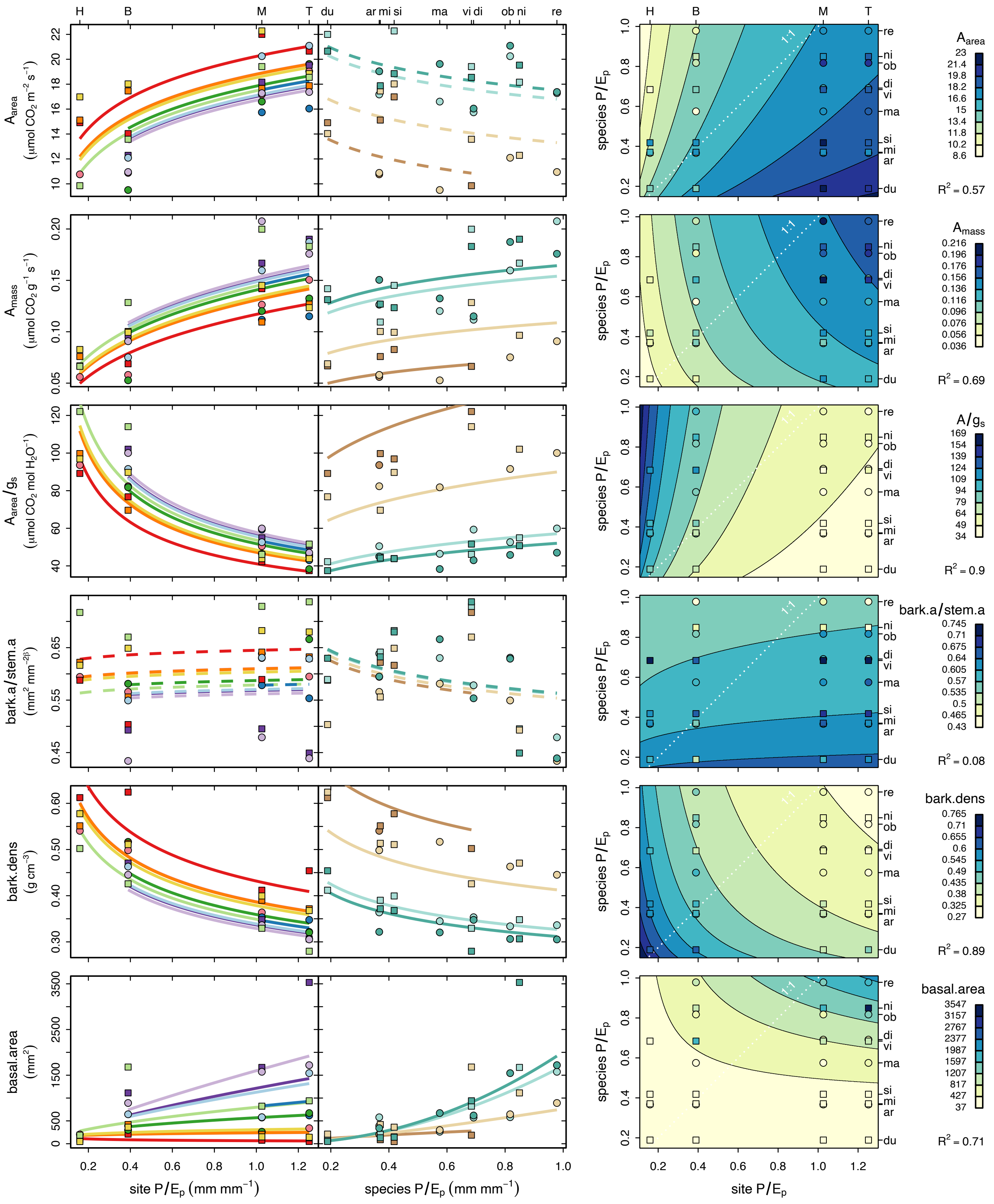

site P/E$_p$ (mm mm^{-1})
species P/E$_p$ (mm mm^{-1})
site P/E$_p$
species P/E$_p$
A$_{area}$ (μmol CO$_2$ m^{-2} s^{-1})
A$_{mass}$ (μmol CO$_2$ g^{-1} s^{-1})
A$_{area}$/g$_s$ (μmol CO$_2$ mol H$_2$O^{-1})
bark.a/stem.a (mm^2 mm$^{-2β}$)
bark.dens (g cm^{-3})
basal.area (mm^2)
R^2 = 0.57
R^2 = 0.69
R^2 = 0.9
R^2 = 0.08
R^2 = 0.89
R^2 = 0.71

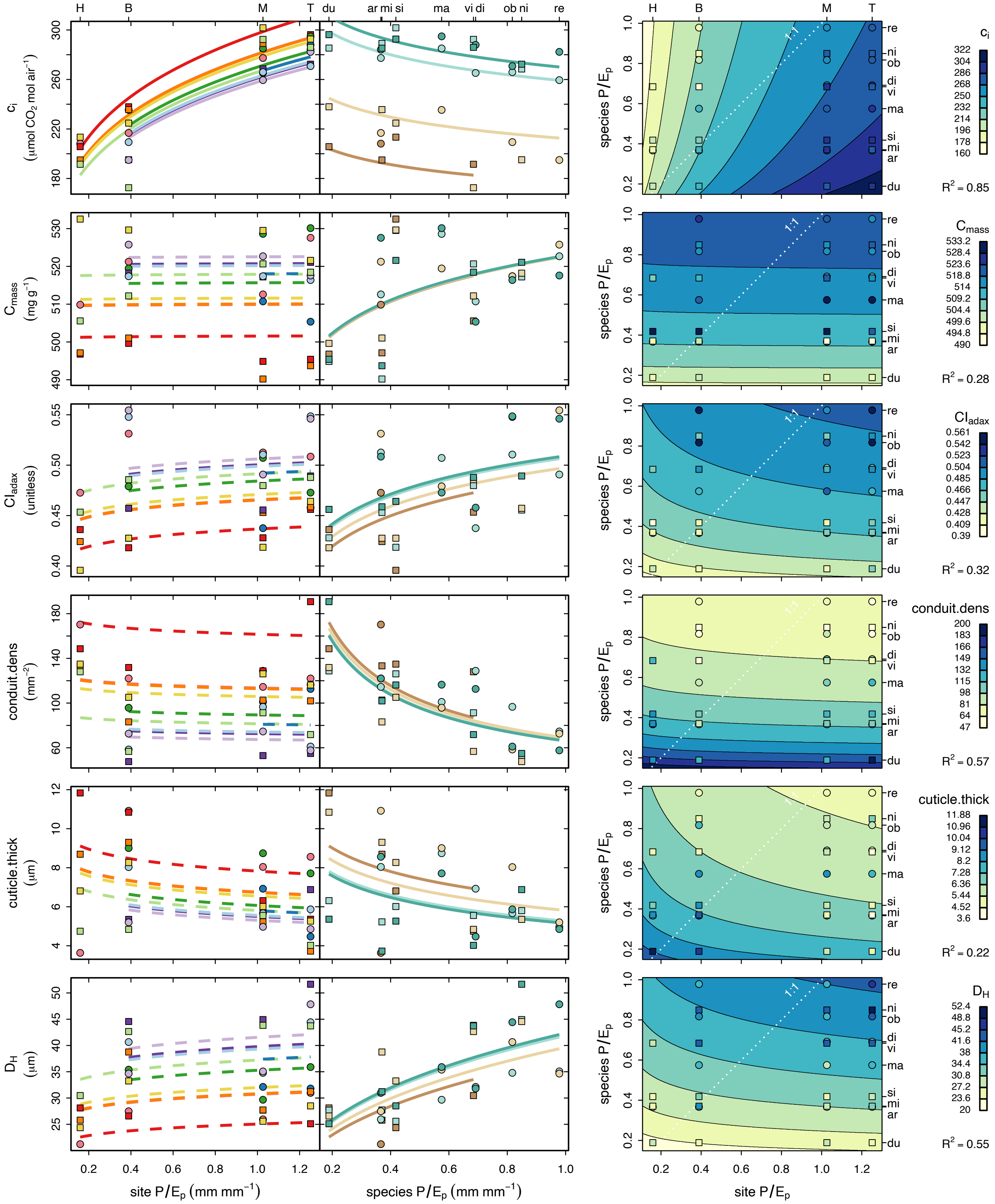
H
B
M
T
du
ar mi si
ma
vi di
ob ni
re
c_i (µmol CO_2 mol air^{-1})
C_{mass} (mg g^{-1})
CI_{adax} (unitless)
conduit.dens (mm^{-2})
cuticle.thick (µm)
D_H (µm)
species P/E_p
1:1
c_i
322
304
286
268
250
232
214
196
178
160
$R^2 = 0.85$
C_{mass}
533.2
528.4
523.6
518.8
514
509.2
504.4
499.6
494.8
490
$R^2 = 0.28$
CI_{adax}
0.561
0.542
0.523
0.504
0.485
0.466
0.447
0.428
0.409
0.39
$R^2 = 0.32$
conduit.dens
200
183
166
149
132
115
98
81
64
47
$R^2 = 0.57$
cuticle.thick
11.88
10.96
10.04
9.12
8.2
7.28
6.36
5.44
4.52
3.6
$R^2 = 0.22$
D_H
52.4
48.8
45.2
41.6
38
34.4
30.8
27.2
23.6
20
$R^2 = 0.55$
site P/E_p (mm mm^{-1})
species P/E_p (mm mm^{-1})
site P/E_p

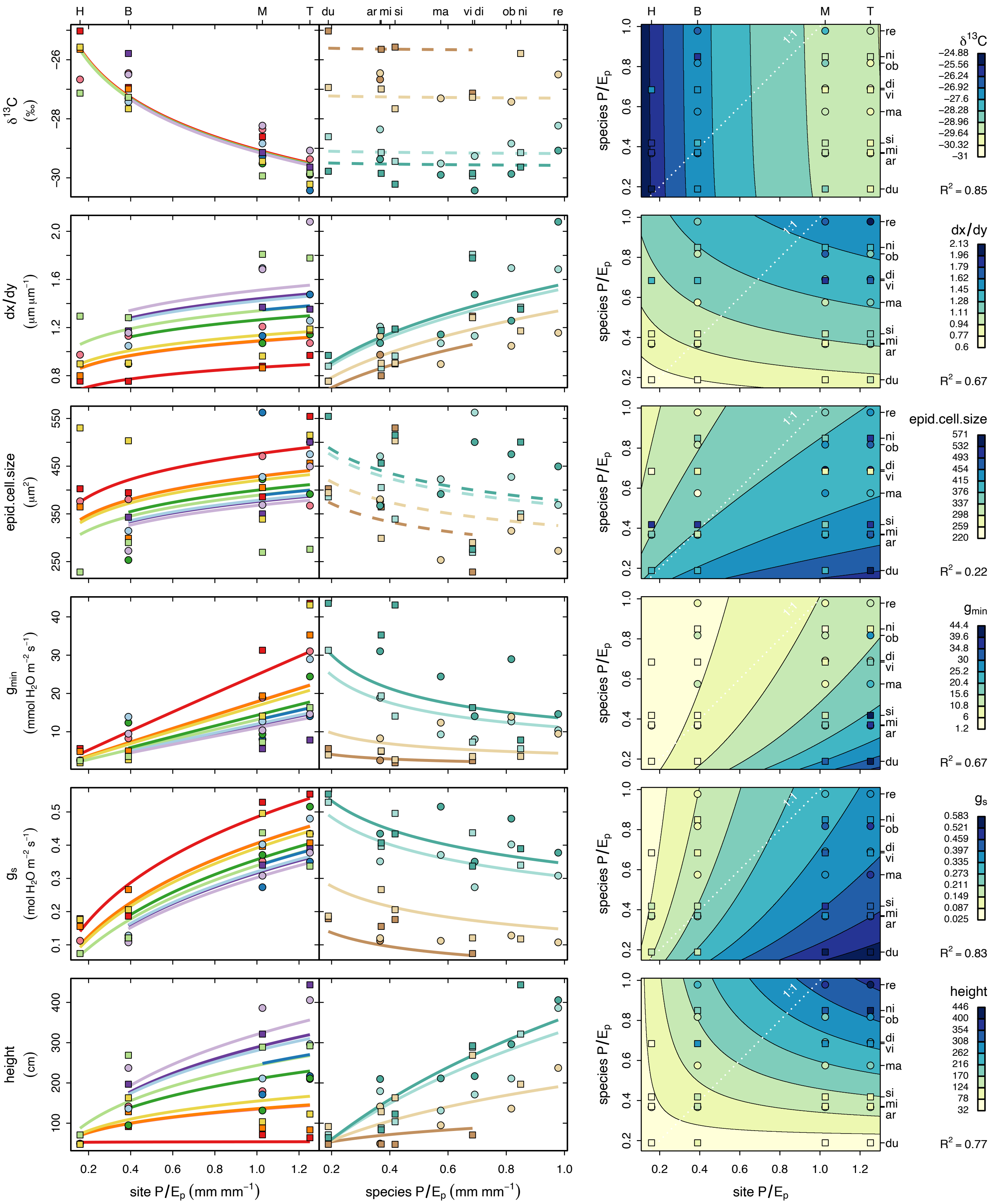

H B M T
du ar mi si ma vi di ob ni re
$\delta^{13}C$ (‰)
dx/dy (μm μm^{-1})
epid.cell.size (μm^2)
g_{min} (mmol H_2O m^{-2} s^{-1})
g_s (mol H_2O m^{-2} s^{-1})
height (cm)
site P/E$_p$ (mm mm^{-1})
species P/E$_p$ (mm mm^{-1})
species P/E$_p$
site P/E$_p$
1:1
R^2 = 0.85
R^2 = 0.67
R^2 = 0.22
R^2 = 0.67
R^2 = 0.83
R^2 = 0.77

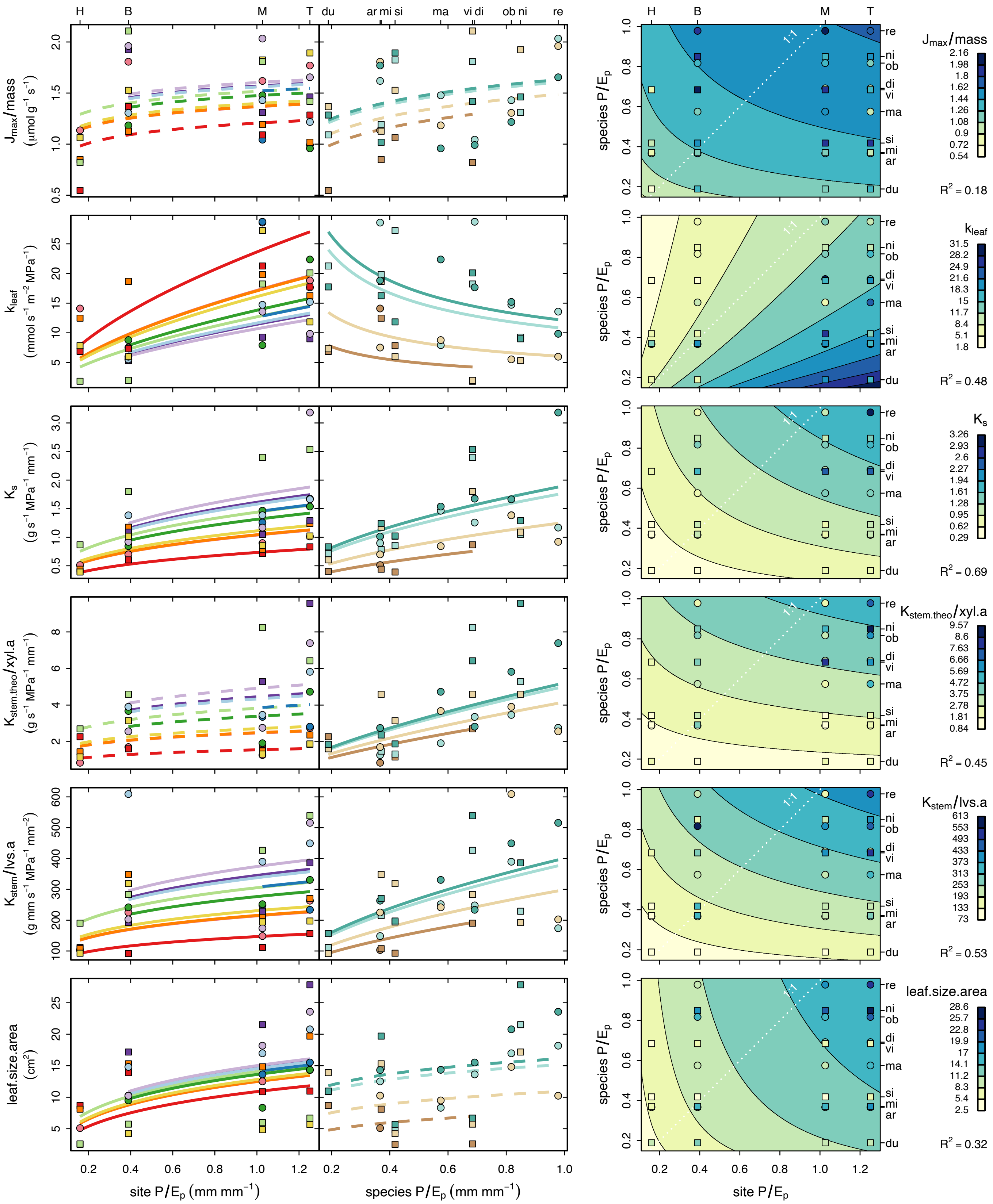

H
B
M
T
du
ar mi si
ma
vi di
ob ni
re
J_{max}/mass (μmol g^{-1} s^{-1})
k_{leaf} (mmol s^{-1} m^{-2} MPa^{-1})
K_s (g s^{-1} MPa^{-1} mm^{-1})
$K_{stem.theo}$/xyl.a (g s^{-1} MPa^{-1} mm^{-1})
K_{stem}/lvs.a (g mm s^{-1} MPa^{-1} mm^{-2})
leaf.size.area (cm^2)
site P/E_p (mm mm^{-1})
species P/E_p (mm mm^{-1})
species P/E_p
site P/E_p
1:1
J_{max}/mass
2.16 1.98 1.8 1.62 1.44 1.26 1.08 0.9 0.72 0.54
R^2 = 0.18
k_{leaf}
31.5 28.2 24.9 21.6 18.3 15 11.7 8.4 5.1 1.8
R^2 = 0.48
K_s
3.26 2.93 2.6 2.27 1.94 1.61 1.28 0.95 0.62 0.29
R^2 = 0.69
$K_{stem.theo}$/xyl.a
9.57 8.6 7.63 6.66 5.69 4.72 3.75 2.78 1.81 0.84
R^2 = 0.45
K_{stem}/lvs.a
613 553 493 433 373 313 253 193 133 73
R^2 = 0.53
leaf.size.area
28.6 25.7 22.8 19.9 17 14.1 11.2 8.3 5.4 2.5
R^2 = 0.32

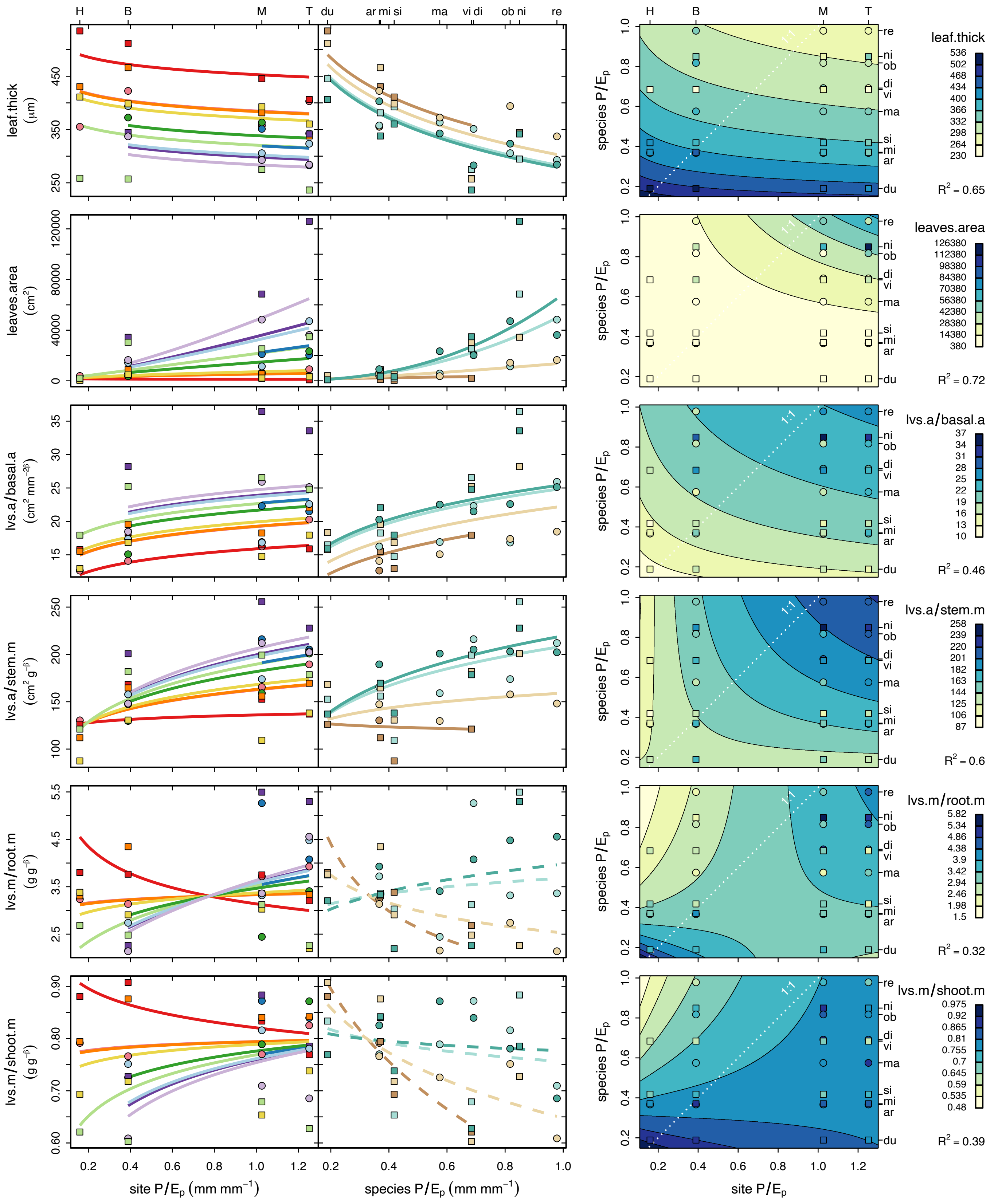

leaf.thick (μm)
leaves.area (cm²)
lvs.a/basal.a
lvs.a/stem.m
lvs.m/root.m (g g⁻¹)
lvs.m/shoot.m (g g⁻¹)
H
B
M
T
du
ar mi si
ma
vi di
ob ni
re
site P/Ep (mm mm⁻¹)
species P/Ep (mm mm⁻¹)
species P/Ep
site P/Ep
1:1
leaf.thick
R² = 0.65
leaves.area
R² = 0.72
lvs.a/basal.a
R² = 0.46
lvs.a/stem.m
R² = 0.6
lvs.m/root.m
R² = 0.32
lvs.m/shoot.m
R² = 0.39

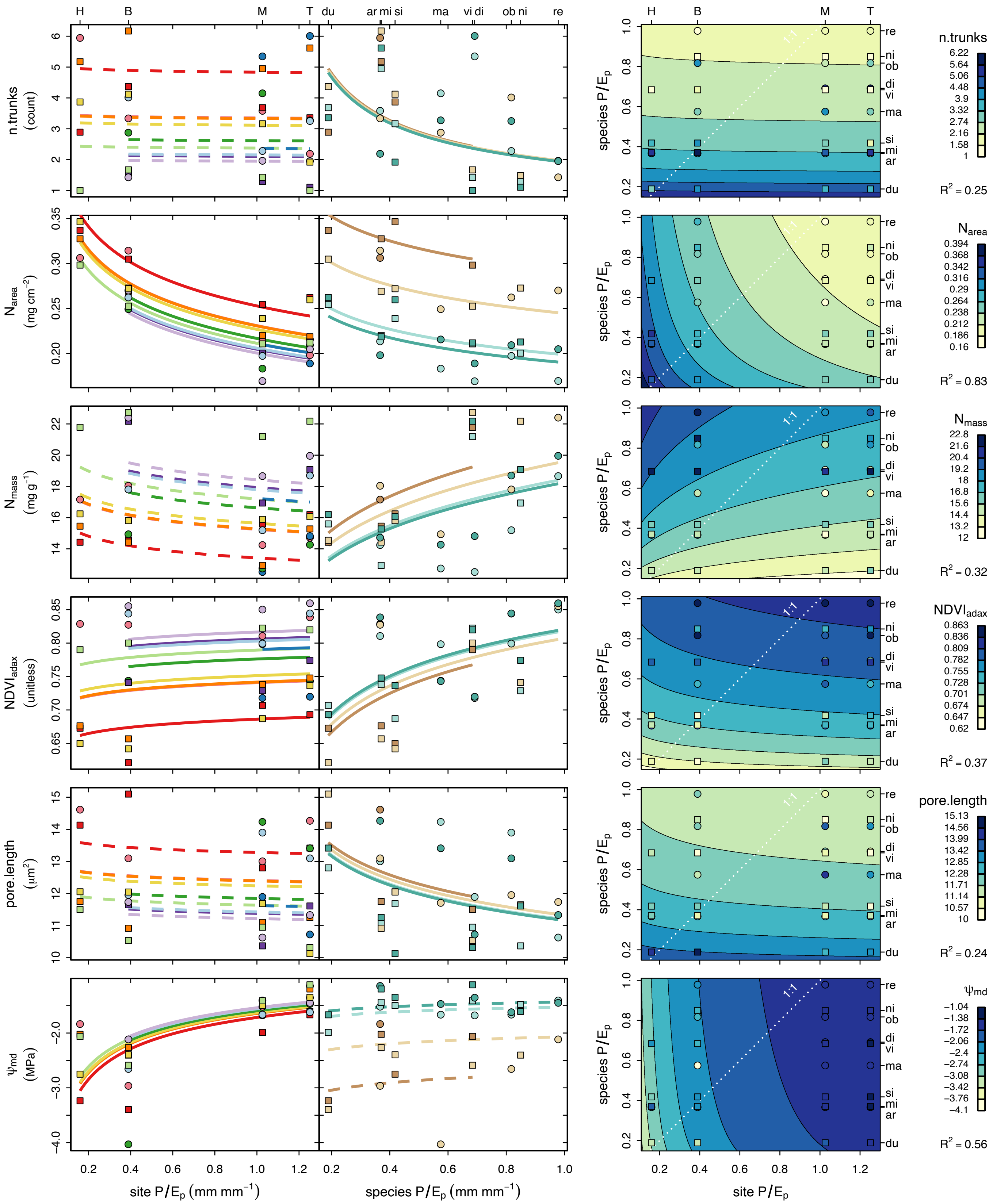

n.trunks
(count)
N_area
(mg cm^-2)
N_mass
(mg g^-1)
NDVI_adax
(unitless)
pore.length
(µm^2)
ψ_md
(MPa)
H B M T
du ar mi si ma vi di ob ni re
site P/E_p (mm mm^-1)
species P/E_p (mm mm^-1)
species P/E_p
site P/E_p
1:1
R^2 = 0.25
R^2 = 0.83
R^2 = 0.32
R^2 = 0.37
R^2 = 0.24
R^2 = 0.56

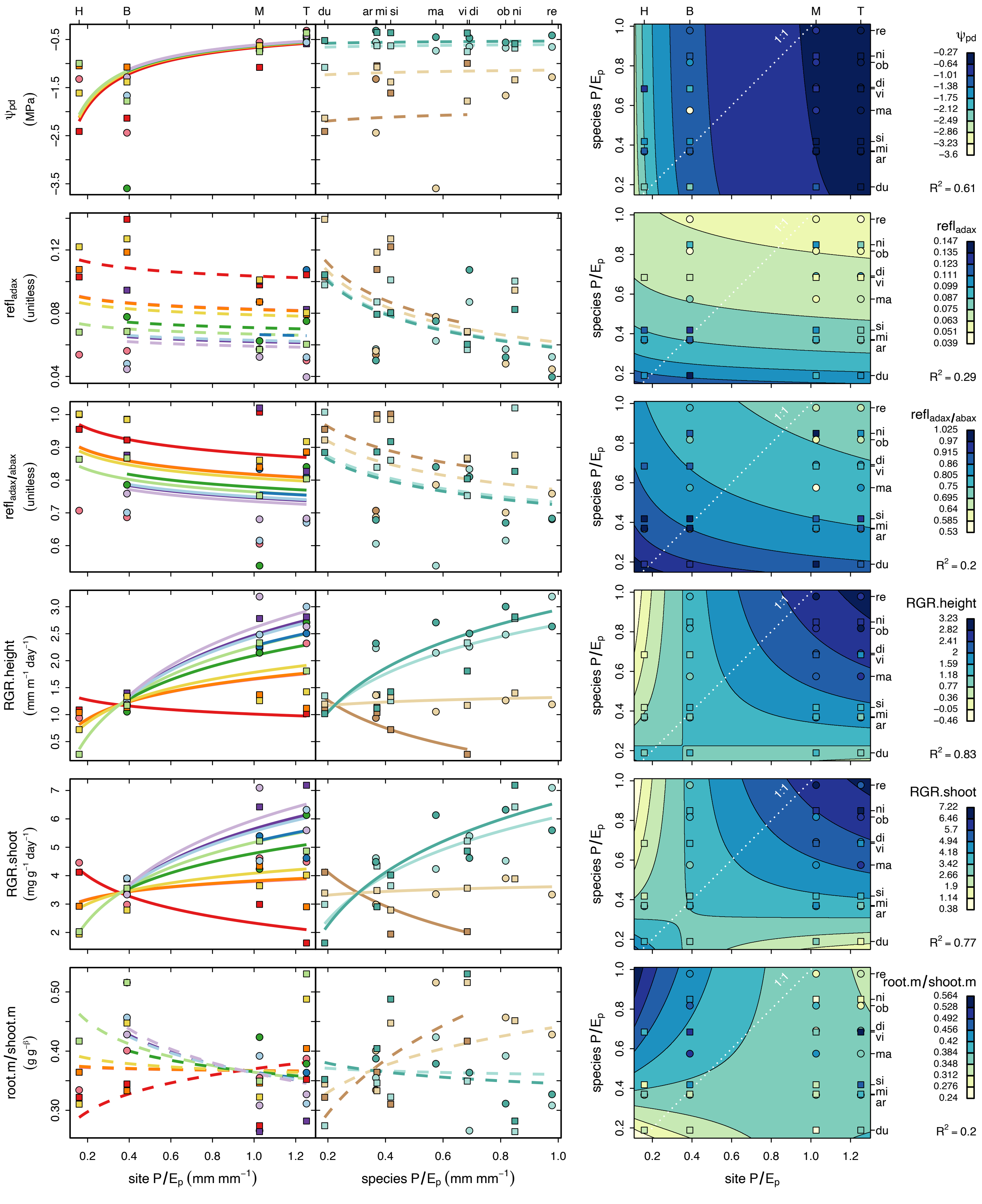
ψpd (MPa)
refl adax (unitless)
refl adax/abax (unitless)
RGR.height (mm m⁻¹ day⁻¹)
RGR.shoot (mg g⁻¹ day⁻¹)
root.m/shoot.m (g g⁻β)
site P/Ep (mm mm⁻¹)
species P/Ep (mm mm⁻¹)
species P/Ep
site P/Ep
H
B
M
T
du
ar
mi
si
ma
vi
di
ob
ni
re
1:1
R² = 0.61
R² = 0.29
R² = 0.2
R² = 0.83
R² = 0.77
R² = 0.2

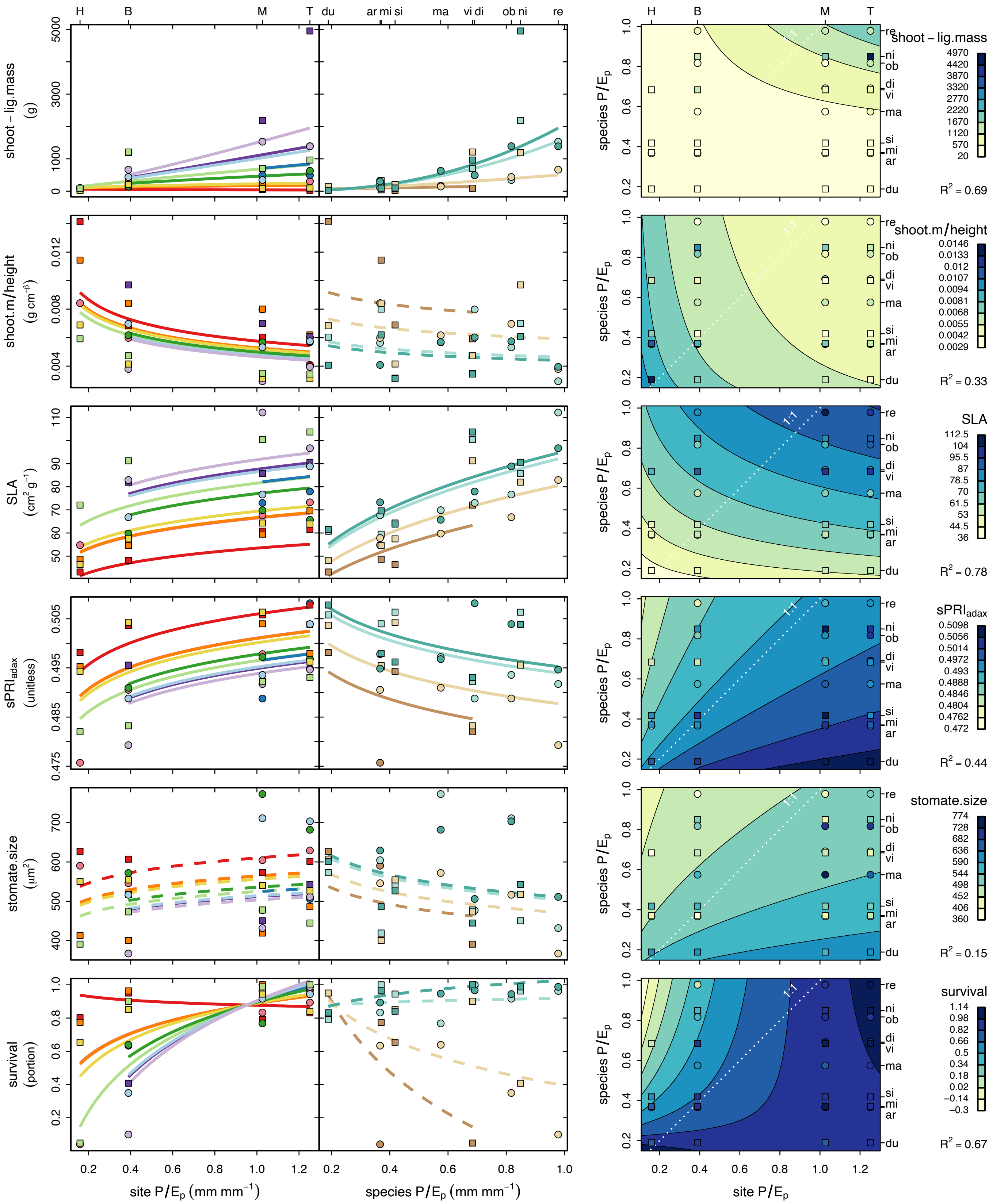

shoot – lig.mass (g)
shoot.m/height (g cm⁻¹)
SLA (cm² g⁻¹)
sPRIadax (unitless)
stomate.size (µm²)
survival (portion)
site P/Ep (mm mm⁻¹)
species P/Ep (mm mm⁻¹)
species P/Ep
site P/Ep
H
B
M
T
du
ar mi si
ma
vi di
ob ni
re
R² = 0.69
R² = 0.33
R² = 0.78
R² = 0.44
R² = 0.15
R² = 0.67
1:1

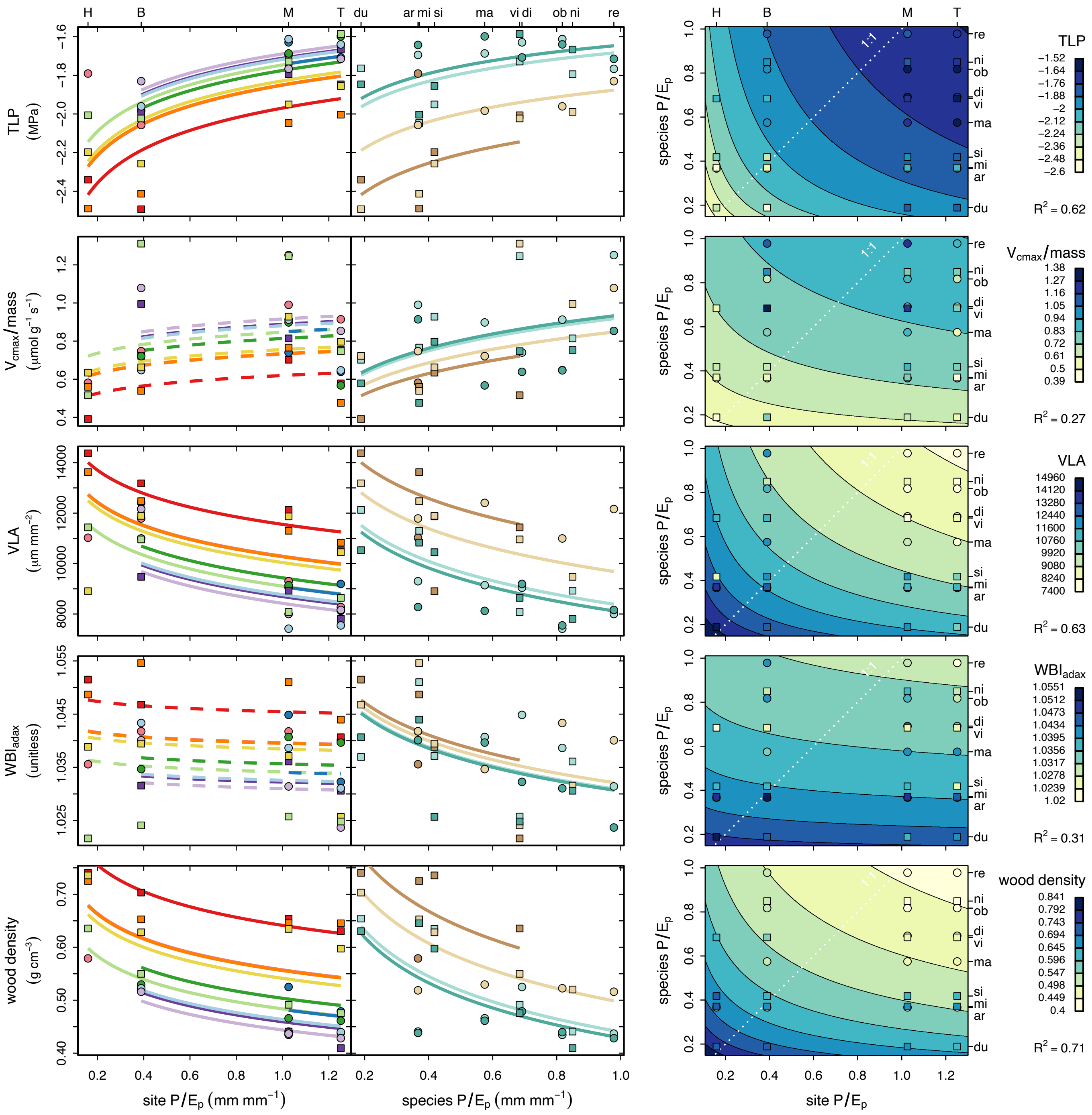

H
B
M
T
du
ar mi si
ma
vi di
ob ni
re
TLP (MPa)
V_{cmax}/mass (μmol g^{-1} s^{-1})
VLA (μm mm^{-2})
WBI_{adax} (unitless)
wood density (g cm^{-3})
site P/E_p (mm mm^{-1})
species P/E_p (mm mm^{-1})
species P/E_p
site P/E_p
1:1
TLP
−1.52
−1.64
−1.76
−1.88
−2
−2.12
−2.24
−2.36
−2.48
−2.6
R^2 = 0.62
V_{cmax}/mass
1.38
1.27
1.16
1.05
0.94
0.83
0.72
0.61
0.5
0.39
R^2 = 0.27
VLA
14960
14120
13280
12440
11600
10760
9920
9080
8240
7400
R^2 = 0.63
WBI_{adax}
1.0551
1.0512
1.0473
1.0434
1.0395
1.0356
1.0317
1.0278
1.0239
1.02
R^2 = 0.31
wood density
0.841
0.792
0.743
0.694
0.645
0.596
0.547
0.498
0.449
0.4
R^2 = 0.71

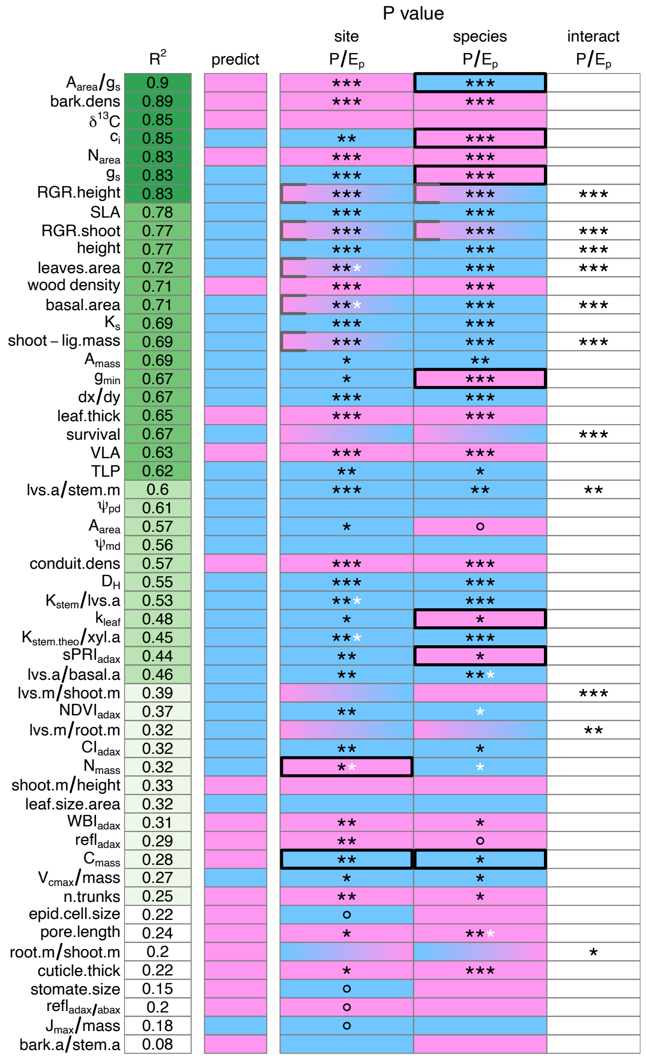

P < 0.05	site	species
agree	32	26
disagree	2	7
disagree at low P/E_p	5	2

Figure S4. Direction, statistical significance, and accord with predictions for trait relationships to species and site P/E_p based on standard (phylogenetically unstructured) regression. Blue indicates traits that increase with P/E_p; pink, traits that decrease with P/E_p. Color gradients indicate a shift in sign as P/E_p increased left to right. Symbols ∘, *,**,*** indicate statistical significance at the 0.1, 0.05, 0.01, and 0.001 levels, corrected for multiple comparisons (white stars indicate significance without correction for multiple comparisons). Thick black walls identify significant relationships counter to theory; gray walls, significant relationships that accord with theory except for the driest sites or species. The bottom table summarizes the numbers of relationships that agree or disagree with predictions at $P < 0.05$. Source data are provided as a Source Data file.

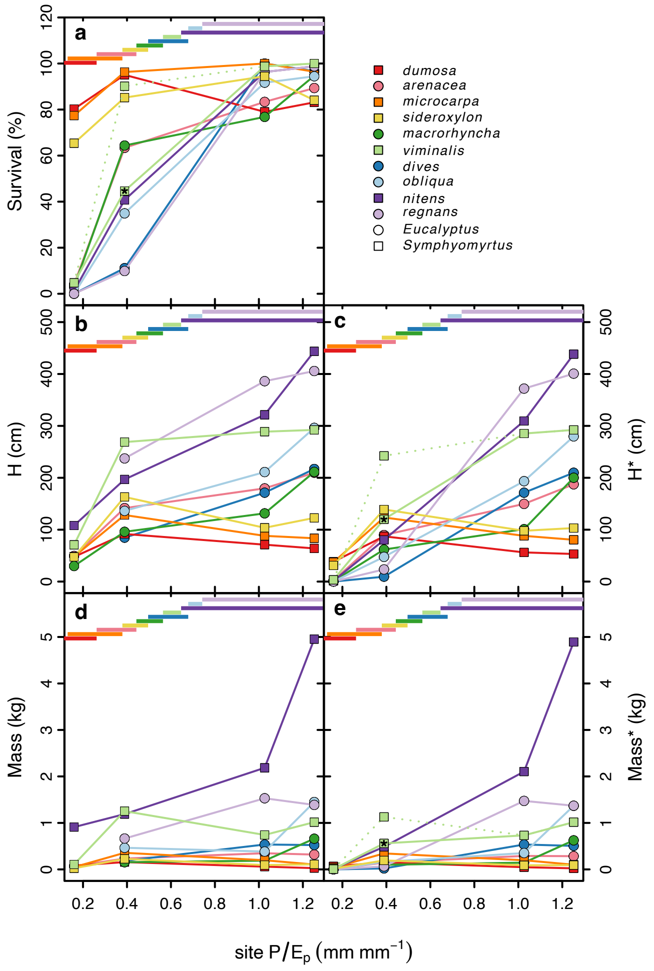

Figure S5. Survival, height growth (H), realized height growth (height times survival, H*), mass growth (M), and realized mass growth (mass times survival, M*) at each garden as a function of site P/E_p. Adaptive cross-over is evident only in realized height growth (**c**), not in survival (**a**), height growth (**b**), mass growth (**d**), or realized mass growth (**e**). The values for *viminalis* in **a,c,e** are both the original data (connected by dotted line) and adjusted for survival (denoted with asterisk) as shown in Fig. 6f. Standard errors and within-site significant differences among means are shown in Fig. 6 for survival, H, and H*, and in Fig. S6 for M and M*. Source data are provided as a Source Data file.

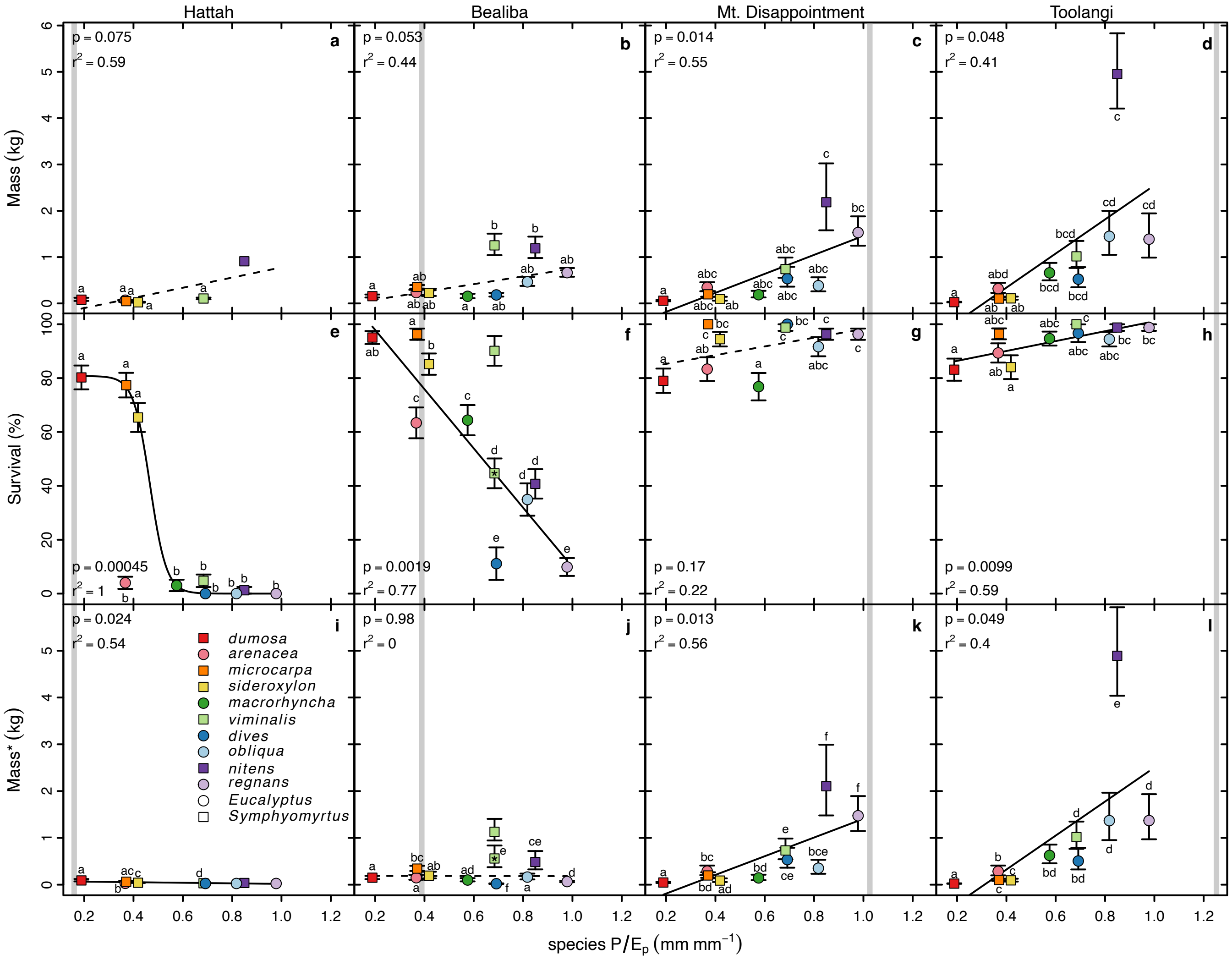

Figure S6. Mass growth (a-d), survival (e-h), and realized mass growth (Mass*; i-j) of species at each garden as functions of species P/E_p. Vertical gray lines indicate site P/E_p. Data are presented as mean ± SE where n plants per species per site was 1 - 7 (mass) and 27 - 84 (survival). See Eqn 1 for Mass* error calculation. Letters indicate significant pairwise differences within sites. Plotting symbols lack letters when comparisons could not be made. Solid regression lines indicate $P < 0.05$. The fit of survival at Hattah (**e**) excludes *arenacea*. Survival of *viminalis* at Bealiba (**f**) is shown as observed and as expected (*) based on regression of survival against species P/E_p of other species there (see text). Mass* of *viminalis* at Bealiba (**j**) follows accordingly. Source data are provided as a Source Data file.

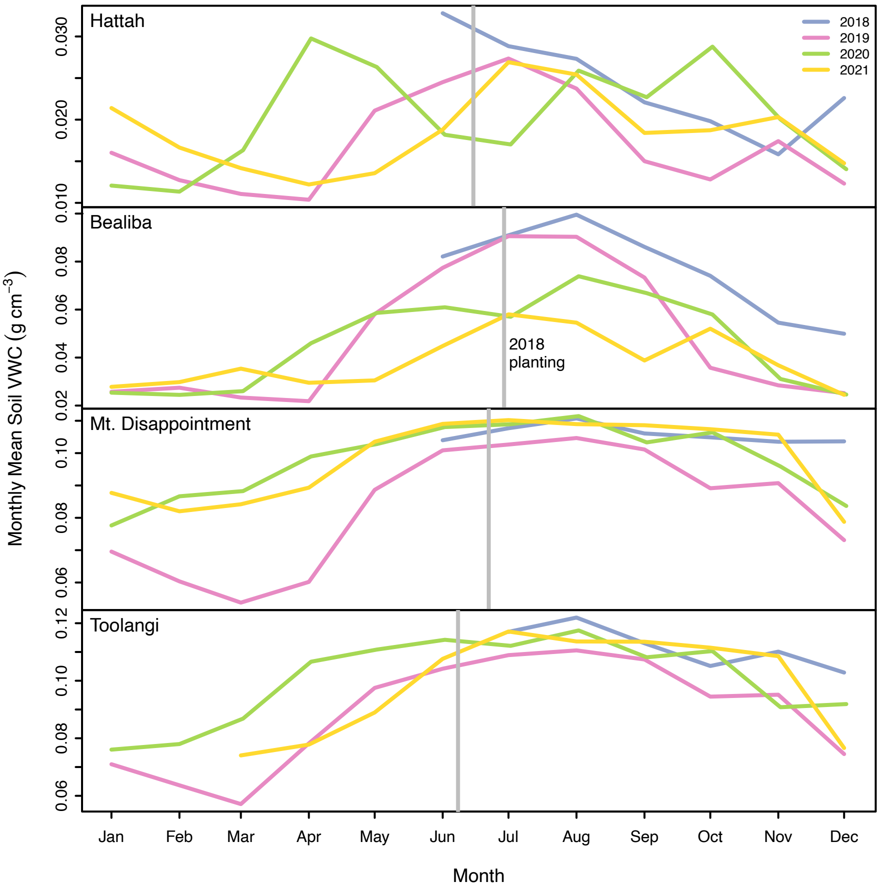

Figure S7 – Monthly means of soil volumetric water content (VWC) over the course of the main experiment at each site. Note the unusually high VWC at Bealiba in 2018, soon after planting the experiment. Source data are provided as a Source Data file.

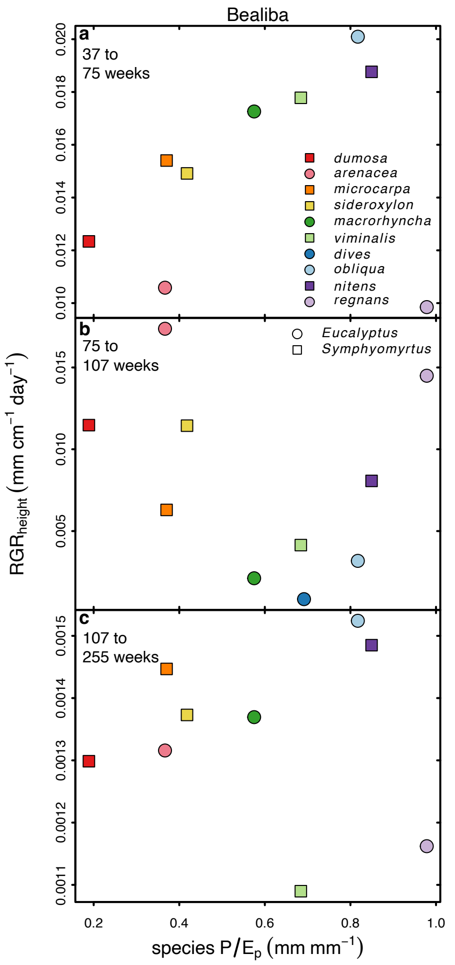

Figure S8 – Relative growth rate of height at second driest site Bealiba between (**a**) the 1st and 2nd harvest, (**b**) 2nd to 3rd harvest, and (**c**) post 3rd harvest. Initially, *Eucalyptus viminalis* performed unexpectedly well, combining high height growth (consistent with its P/E_p) with anomalously high survival (Figs. 6 and S4). However, later in the study, its height growth plunged relative to most other species, indicating that its unexpected performance was short-lived. Source data are provided as a Source Data file.

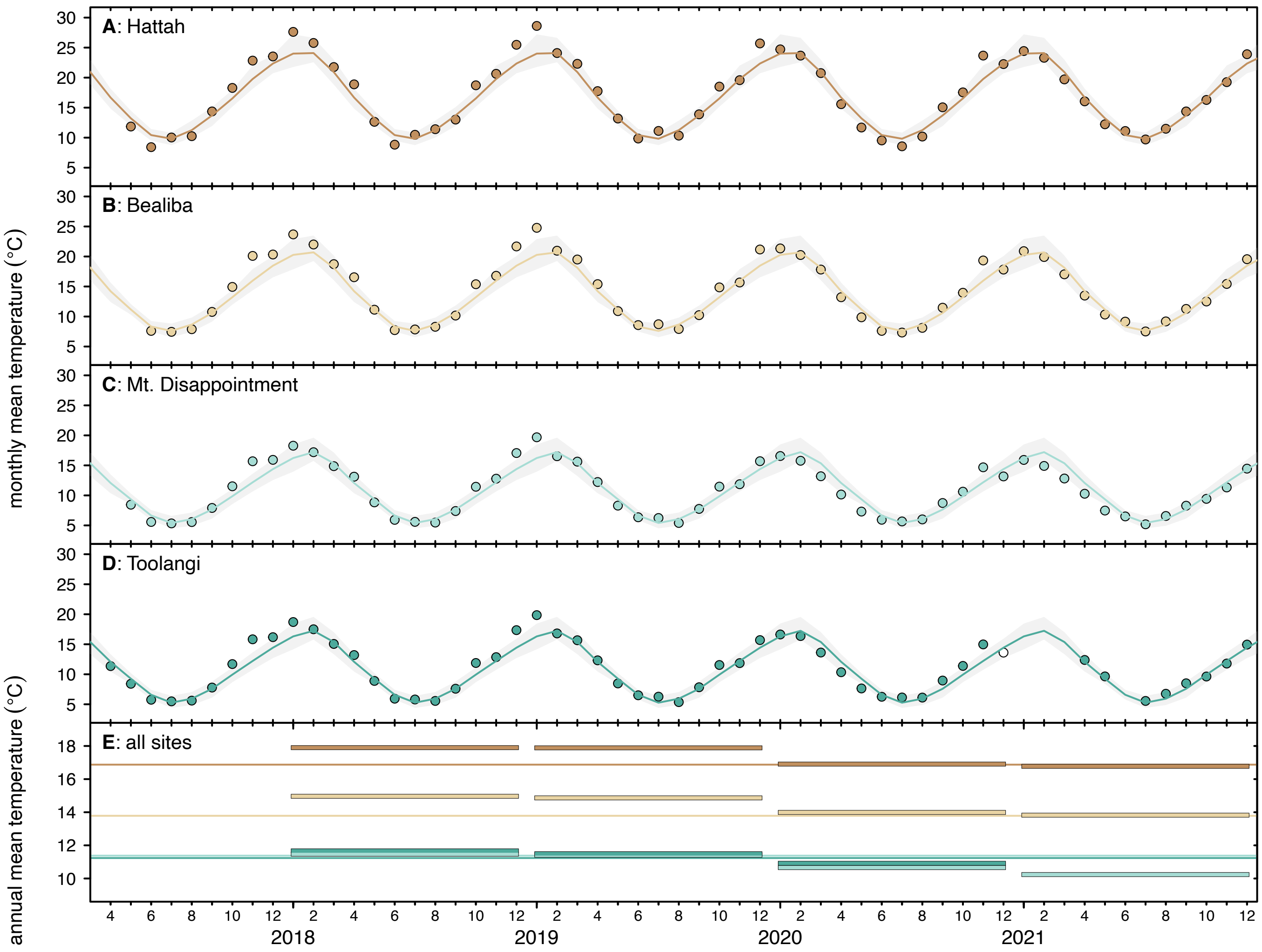

Figure S9. **A-D**: Monthly mean temperatures (dots) during study years compared with means across the 1970-2000 reference period (curves ± 90% confidence intervals), color- coded by site. **E**: Annual mean temperatures (bars) during study period at each site compared those for the reference period (lines). The open symbol at December 2020 in panel D indicates a partial month (21 days) of data before weather station vandalism, followed by missing data prior to repair. Source data are provided as a Source Data file.

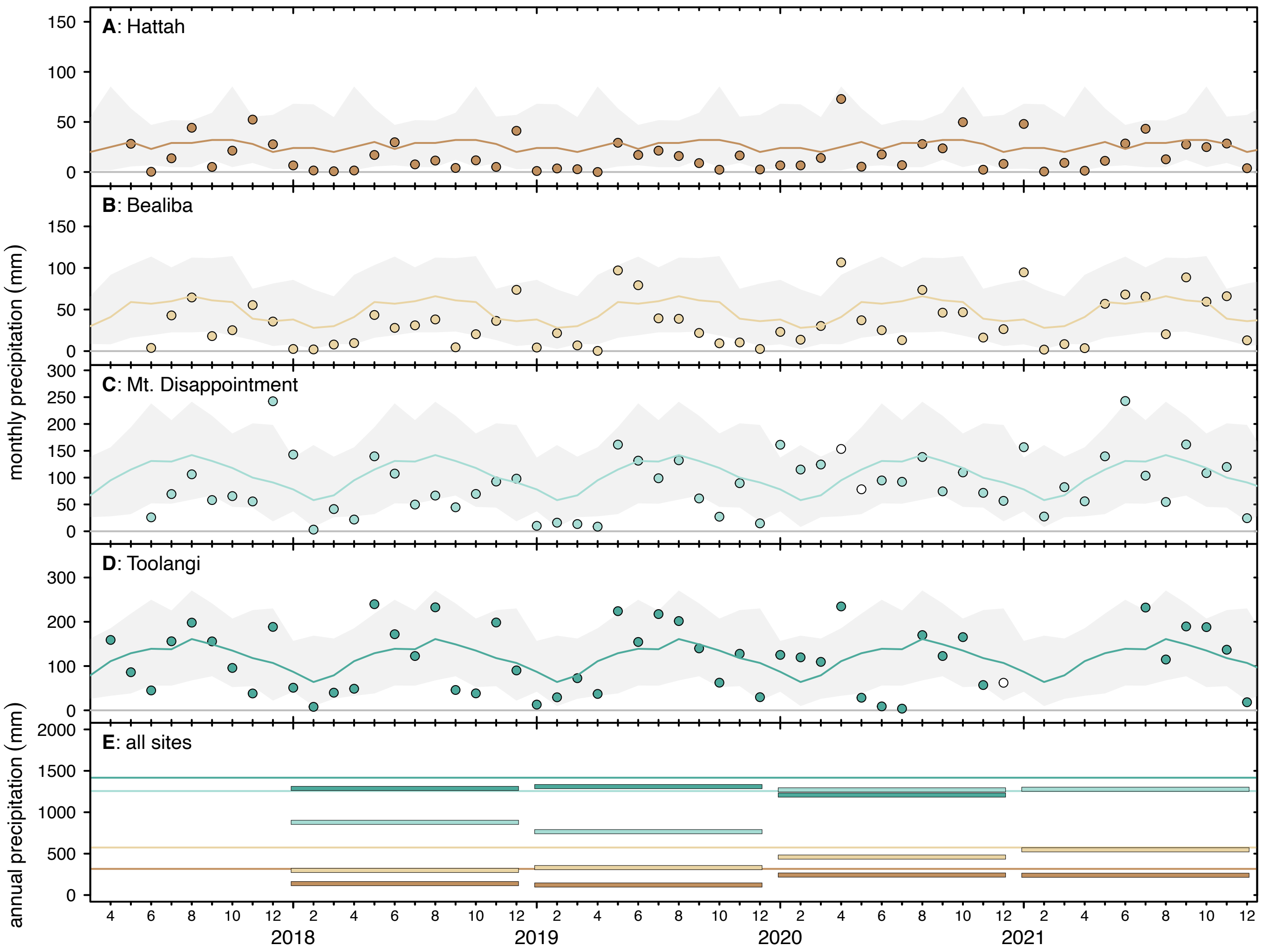

Figure S10. **A-D**: Monthly mean precipitation (dots) during study years compared with means across the 1970-2000 reference period (curves ± 90% confidence intervals), color-coded by site. **E**: Annual precipitation (bars) during study period at each site compared to means in the reference period (lines). Open symbols in panels C and D indicate partial totals due to weather station malfunction or vandalism, respectively. Source data are provided as a Source Data file.

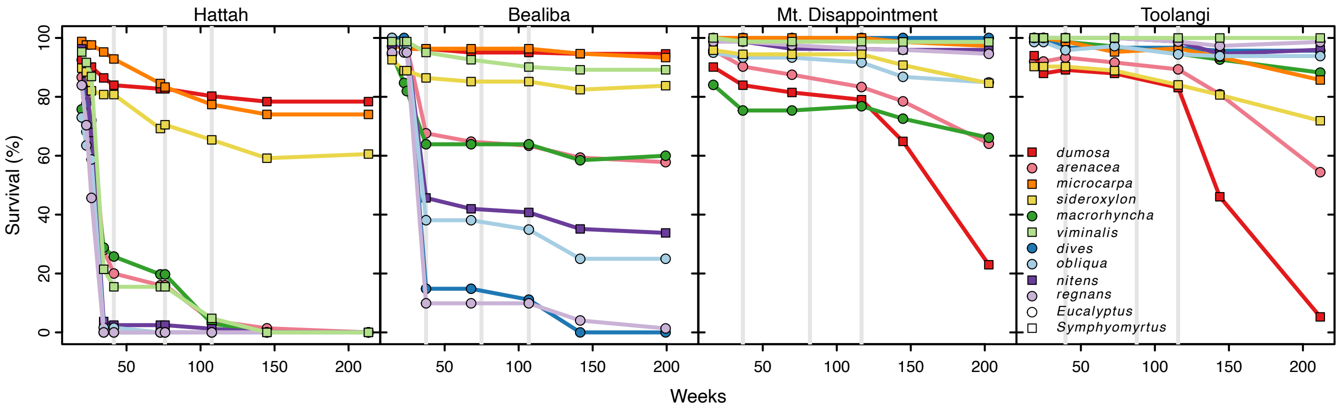

Figure S11. Survival of plants designated for the third harvest as a function of weeks since planting. Survival was measured at or near the times of harvest (vertical lines), prior to the first harvest (especially at drier sites) and twice after the third harvest. Survival after the third harvest utilizes plants left in the ground. Survival percent occasionally increased slightly due to either error or recovery of plants that appeared dead. Source data are provided as a Source Data file.

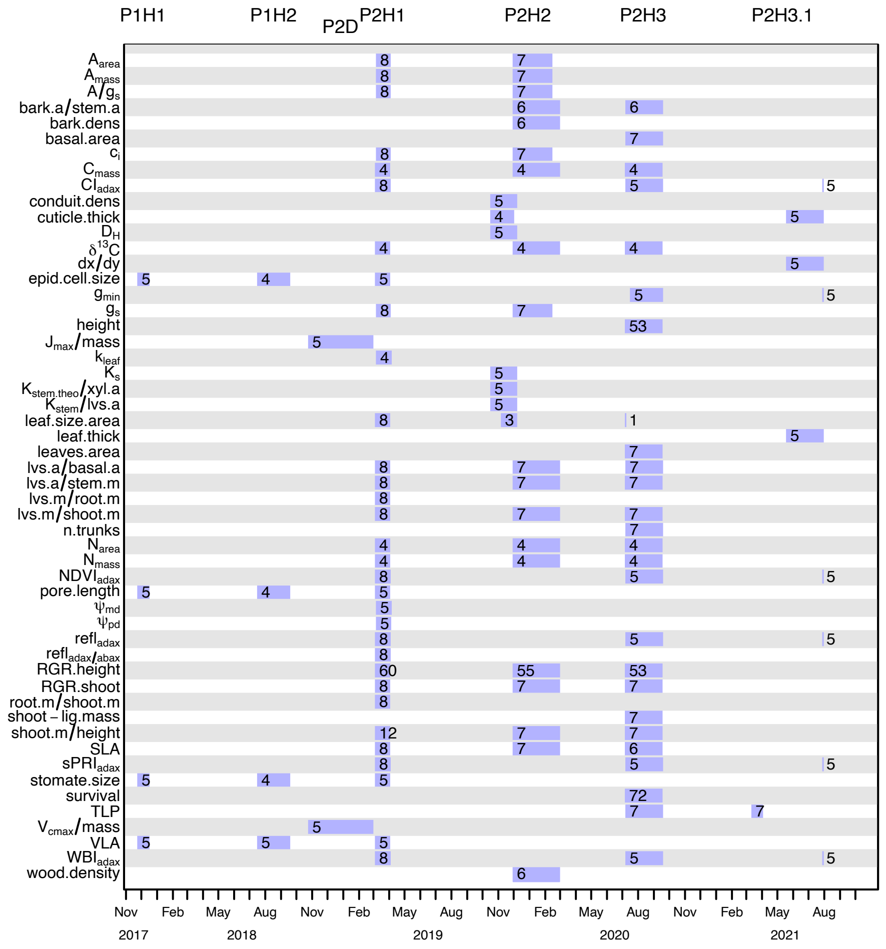

Figure S12. Timing of trait measurements over the course of the pilot and main experiments. Shading indicates period of data collection; number is mean number of replicates per species per site per period. P1 = pilot experiment; P2 = main experiment; H1, H2, and so forth indicate the harvest/field campaign during which the data were gathered. "D" represents measurements from a parallel drought experiment at each site.

Table S1. Mean annual precipitation (P), pan evaporation (E_p), and P/E_p for each study species averaged across its range.

Species	P (mm)	E_p (mm)	P/E_p
Subgenus *Symphyomyrtus*			
E. dumosa	350	1930	0.19
E. microcarpa	594	1690	0.37
E. sideroxylon	681	1693	0.42
E. viminalis	855	1324	0.68
E. nitens	1014	1219	0.85
Subgenus *Eucalyptus*			
E. arenacea	546	1526	0.37
E. macrorhyncha	797	1453	0.58
E. dives	884	1315	0.69
E. obliqua	954	1251	0.82
E. regnans	1042	1107	0.98

Table S2. Common-garden locations, elevations, climatic parameters, and depths to water table (DTWT).

Garden	Location	Elevation (m)	DTWT (m)	P (mm)	E_p (mm)	P/E_p
Hattah	34.74 S, 142.25 E	42	5.8	316	1983	0.16
Bealiba	36.73 S, 143.61 E	280	4.8	574	1475	0.39
Mt. Disappointment	36.39 S, 145.14 E	650	23.8	1256	1224	1.03
Toolangi	36.49 S, 145.57 E	688	42.2	1417	1132	1.25

Table S3: Results of phylogenetically structured mixed model regression of traits, transformed as indicated for normalization. Fixed effects were log-transformed site P/E_p, species P/E_p and their interaction. Random effects were site, species (sp), phylogeny (phyl) and phylogenetic attraction (attr). Random effect columns indicate: ***= $p < 0.001$, **= $p < 0.01$, *= $p < 0.05$, ◦ = $p < 0.1$ and + = $p < 0.5$. All random effects with symbols were retained. Fixed effect columns give coefficients with significance symbolized as above except for +. Standard error and exact p values follow coefficients. The p values for species P/E_p and site P/E_p were adjusted with Yekutieli-Benjamini-Hochberg procedure. All fixed effects except interaction were always kept. Interaction was kept when $p < 0.05$. R^2_{tot} is the likelihood-based R^2 for the full model while $R^2_{part\ fix}$ and $R^2_{part\ rand}$ are likelihood-based partial R^2 values for fixed and random effects. R^2_{fix} is the R^2 calculated from just the fixed effects prediction of the full model. Unit exponents β control for allometric scaling of trait ratios (see Source Data).

Trait	Units	Trans	site	sp	phyl	attr	Intercept	site P/E_p	species P/E_p	Interact	R^2_{tot}	$R^2_{part\ fix}$	$R^2_{part\ rand}$	R^2_{fix}
A_{area}	μmol CO_2 m^{-2} s^{-1}			+		◦	16.821***<br>±0.950<br>p =4.321×10^{-70}	3.665***<br>±0.695<br>p =4.853×10^{-7}	-1.761<br>±0.982<br>p =0.101		0.634	0.34	0.14	0.57
A_{area}/g_s	μmol CO_2 mol H_2O^{-1}	log	+			+	4.057***<br>±0.054<br>p =0	-0.443***<br>±0.051<br>p =1.063×10^{-16}	0.194***<br>±0.030<br>p =9.632×10^{-10}		0.929	0.652	0.288	0.896
A_{mass}	μmol CO_2 g^{-1} s^{-1}	sqrt		+	+	+	0.398***<br>±0.018<br>p =1.803×10^{-111}	0.067***<br>±9.883×10^{-3}<br>p =6.839×10^{-11}	0.041**<br>±0.014<br>p =0.007		0.728	0.447	0.134	0.675
bark.a/stem.a	mm^2 $mm^{-2\beta}$	log	*	***			-0.592***<br>±0.067<br>p =1.022×10^{-18}	0.027<br>±0.022<br>p =0.252	-0.105<br>±0.081<br>p =0.235		0.682	0.077	0.653	0.076
bark.dens	g cm^{-3}	log	***	*			-1.118***<br>±0.039<br>p =4.770×10^{-182}	-0.229***<br>±0.033<br>p =2.423×10^{-11}	-0.173***<br>±0.028<br>p =1.984×10^{-9}		0.924	0.528	0.324	0.887
basal.area	mm^2	log	+	**		+	7.450***<br>±0.307<br>p =3.963×10^{-130}	1.166***<br>±0.277<br>p =6.595×10^{-5}	1.972***<br>±0.269<br>p =2.601×10^{-12}	0.938***<br>±0.173<br>p =5.832×10^{-8}	0.81	0.645	0.346	0.693
c_i	μmol CO_2 mol air^{-1}	log	*				5.554***<br>±0.026<br>p =0	0.196***<br>±0.024<br>p =2.886×10^{-15}	-0.078***<br>±0.020<br>p =1.803×10^{-4}		0.863	0.492	0.112	0.844
C_{mass}	mg g^{-1}	log		***			6.258***<br>±9.171×10^{-3}<br>p =0	1.286×10^{-3}<br>±2.354×10^{-3}<br>p =0.608	0.023◦<br>±0.012<br>p =0.064		0.653	0.106	0.519	0.276
CI_{adax}	unitless	exp		***		◦	1.656***<br>±0.026	0.020<br>±0.012	0.060◦<br>±0.031		0.615	0.159	0.435	0.315

continued

Trait	Units	Trans	site	sp	phyl	attr	Intercept	site P/E_p	species P/E_p	Interact	R^2_{tot}	$R^2_{part\ fix}$	$R^2_{part\ rand}$	R^2_{fix}
							p =0	p =0.107	p =0.070					
conduit.dens	mm^{-2}	log	*	*	+		4.182***<br>±0.127<br>p =1.944×10^{-236}	-0.081<br>±0.081<br>p =0.348	-0.561***<br>±0.102<br>p =1.627×10^{-7}		0.667	0.31	0.227	0.553
cuticle.thick	μm	sqrt				+	2.243***<br>±0.138<br>p =1.901×10^{-59}	-0.018<br>±0.128<br>p =0.896	-0.416***<br>±0.110<br>p =3.323×10^{-4}		0.283	0.27	0.075	0.177
D_H	μm	log	*	*	+		3.747***<br>±0.078<br>p =0	0.077<br>±0.049<br>p =0.144	0.333***<br>±0.060<br>p =1.265×10^{-7}		0.642	0.339	0.199	0.54
$\delta^{13}C$	‰	log	+	+			-3.372***<br>±9.049×10^{-3}<br>p =0	-0.067***<br>±6.403×10^{-3}<br>p =2.203×10^{-24}	-1.993×10^{-3}<br>±9.410×10^{-3}<br>p =0.848		0.86	0.332	0.09	0.846
dx/dy	μm μm^{-1}	log		*			0.414***<br>±0.057<br>p =6.156×10^{-13}	0.140***<br>±0.028<br>p =1.982×10^{-6}	0.328***<br>±0.072<br>p =1.341×10^{-5}		0.712	0.597	0.136	0.665
epid.cell.size	μm^2	log		*		○	5.915***<br>±0.089<br>p =0	0.107°<br>±0.053<br>p =0.062	-0.139<br>±0.102<br>p =0.209		0.351	0.127	0.171	0.21
g_{min}	mmol H_2O m^{-2} s^{-1}	log				**	2.332***<br>±0.187<br>p =7.754×10^{-36}	0.989***<br>±0.174<br>p =6.447×10^{-8}	-0.614***<br>±0.142<br>p =4.220×10^{-5}		0.743	0.544	0.22	0.665
g_s	mol H_2O m^{-2} s^{-1}	sqrt	+	+		+	0.553***<br>±0.030<br>p =7.585×10^{-76}	0.165***<br>±0.026<br>p =1.326×10^{-9}	-0.077***<br>±0.021<br>p =5.243×10^{-4}		0.87	0.398	0.244	0.825
height	cm	sqrt	+	**	+	+	18.539***<br>±2.221<br>p =7.085×10^{-17}	5.489***<br>±0.851<br>p =7.018×10^{-10}	6.670***<br>±0.912<br>p =2.845×10^{-12}	3.252***<br>±0.581<br>p =2.123×10^{-8}	0.834	0.697	0.279	0.755
$J_{max}/mass$	μmol g^{-1} s^{-1}		**	**			1.563***<br>±0.185<br>p =3.570×10^{-17}	0.227<br>±0.128<br>p =0.103	0.162<br>±0.177<br>p =0.384		0.417	0.091	0.287	0.13
k_{leaf}	mmol s^{-1} m^{-2} MPa^{-1}	log					2.358***<br>±0.149<br>p =2.210×10^{-16}	0.598***<br>±0.119<br>p =5.236×10^{-5}	-0.483*<br>±0.178<br>p =0.019		0.476			0.476
K_s	g s^{-1} MPa^{-1} mm^{-1}	log		*		○	0.550***<br>±0.118<br>p =2.952×10^{-6}	0.376***<br>±0.076<br>p =2.249×10^{-6}	0.503***<br>±0.130<br>p =2.439×10^{-4}		0.735	0.396	0.143	0.689
$K_{stem.theo}/xyl.a$	g s^{-1} MPa^{-1} mm^{-1}	log	+	+	+		1.635***<br>±0.200<br>p =3.226×10^{-16}	0.223<br>±0.129<br>p =0.110	0.755***<br>±0.178<br>p =5.629×10^{-5}		0.506	0.288	0.1	0.445

continued

Trait	Units	Trans	site	sp	phyl	attr	Intercept	site P/E_p	species P/E_p	Interact	R^2_{tot}	$R^2_{part\ fix}$	$R^2_{part\ rand}$	R^2_{fix}
$K_{stem}/lvs.a$	g mm s^{-1} MPa^{-1} mm^{-2}	log	+	+			5.917***<br>±0.144<br>$p=0$	0.289*<br>±0.110<br>$p=0.015$	0.532***<br>±0.142<br>$p=3.857\times10^{-4}$		0.558	0.311	0.067	0.523
$leaf.size.area$	cm^2	sqrt		***		+	3.945***<br>±0.362<br>$p=1.329\times10^{-27}$	0.530***<br>±0.099<br>$p=3.813\times10^{-7}$	0.452<br>±0.453<br>$p=0.348$		0.731	0.331	0.606	0.31
$leaf.thick$	μm			*			284.120***<br>±16.062<br>$p=5.146\times10^{-70}$	-24.957**<br>±7.569<br>$p=0.002$	-98.475***<br>±20.025<br>$p=2.901\times10^{-6}$		0.712	0.493	0.167	0.651
$leaves.area$	cm^2	log		**		***	10.902***<br>±0.326<br>$p=2.428\times10^{-245}$	1.542***<br>±0.285<br>$p=2.578\times10^{-7}$	2.362***<br>±0.338<br>$p=2.423\times10^{-11}$	1.124***<br>±0.212<br>$p=1.146\times10^{-7}$	0.814	0.657	0.345	0.713
$lvs.a/basal.a$	cm^2 $mm^{-2\beta}$	sqrt		***	+	+	5.076***<br>±0.235<br>$p=9.032\times10^{-104}$	0.304***<br>±0.070<br>$p=4.570\times10^{-5}$	0.759**<br>±0.220<br>$p=0.001$		0.687	0.374	0.419	0.439
$lvs.a/stem.m$	cm^2 $g^{-\beta}$	sqrt		***		*	14.393***<br>±0.431<br>$p=3.597\times10^{-244}$	1.742***<br>±0.307<br>$p=6.447\times10^{-8}$	1.627**<br>±0.515<br>$p=0.003$	0.854**<br>±0.268<br>$p=0.001$	0.744	0.502	0.353	0.603
$lvs.m/root.m$	g $g^{-\beta}$	log					1.293***<br>±0.074<br>$p=2.664\times10^{-17}$	0.388**<br>±0.105<br>$p=0.002$	0.090<br>±0.093<br>$p=0.374$	0.354**<br>±0.111<br>$p=0.003$	0.316			0.316
$lvs.m/shoot.m$	g $g^{-\beta}$	exp		***			2.125***<br>±0.067<br>$p=2.021\times10^{-223}$	0.162***<br>±0.041<br>$p=2.063\times10^{-4}$	-0.088<br>±0.085<br>$p=0.336$	0.155***<br>±0.043<br>$p=2.953\times10^{-4}$	0.607	0.352	0.355	0.368
$n.trunks$	count	log		***	+	+	0.614*<br>±0.274<br>$p=0.025$	-0.098<br>±0.071<br>$p=0.206$	-0.635*<br>±0.278<br>$p=0.035$		0.61	0.137	0.478	0.222
N_{area}	mg cm^{-2}	sqrt		+		**	0.449***<br>$\pm8.198\times10^{-3}$<br>$p=0$	-0.049***<br>$\pm6.863\times10^{-3}$<br>$p=8.164\times10^{-12}$	-0.030***<br>$\pm7.188\times10^{-3}$<br>$p=6.595\times10^{-5}$		0.866	0.533	0.208	0.829
N_{mass}	mg g^{-1}	log		***		*	2.914***<br>±0.066<br>$p=0$	-0.044<br>±0.029<br>$p=0.165$	0.192*<br>±0.079<br>$p=0.025$		0.645	0.167	0.475	0.314
$NDVI_{adax}$	unitless	exp		***		+	2.260***<br>±0.057<br>$p=0$	0.033°<br>±0.016<br>$p=0.054$	0.161*<br>±0.071<br>$p=0.037$		0.734	0.195	0.579	0.365
$pore.length$	μm^2	log		***	+		2.396***<br>±0.049<br>$p=0$	-0.023<br>±0.015<br>$p=0.165$	-0.138**<br>±0.040<br>$p=0.001$		0.495	0.214	0.336	0.196
ψ_{md}	MPa	log	***	*			-0.420***	0.286**	0.092		0.733	0.164	0.393	0.555

continued

Trait	Units	Trans	site	sp	phyl	attr	Intercept	site P/E_p	species P/E_p	Interact	R^2_{tot}	$R^2_{part\ fix}$	$R^2_{part\ rand}$	R^2_{fix}
							±0.113	±0.095	±0.081					
							p =2.054×10^{-4}	p =0.005	p =0.294					
ψ_{pd}	MPa	log	*	+		+	0.517*	0.576**	0.124		0.768	0.159	0.412	0.596
							±0.212	±0.190	±0.124					
							p =0.015	p =0.004	p =0.348					
$refl_{adax}$	unitless	log		***		+	-2.819***	-0.067	−0.322°		0.701	0.149	0.578	0.29
							±0.138	±0.037	±0.173					
							p =2.291×10^{-92}	p =0.101	p =0.088					
$refl_{adax/abax}$	unitless	log		***	*		-0.274***	−0.045°	-0.037		0.558	0.126	0.445	0.14
							±0.080	±0.021	±0.055					
							p =5.629×10^{-4}	p =0.052	p =0.528					
$RGR.height$	cm cm^{-1} day^{-1}			+		+	2.583***	1.313***	0.886***	0.827***	0.841	0.714	0.078	0.824
							±0.122	±0.142	±0.134	±0.131				
							p =6.230×10^{-99}	p =7.443×10^{-19}	p =2.787×10^{-10}	p =3.118×10^{-10}				
$RGR.shoot$	g g^{-1} day^{-1}						6.009***	2.539***	2.206***	2.141***	0.771			0.771
							±0.230	±0.326	±0.291	±0.345				
							p =3.208×10^{-22}	p =5.621×10^{-8}	p =8.068×10^{-8}	p =7.765×10^{-7}				
$root.m/shoot.m$	g g$^{-\beta}$	sqrt	○	+			0.604***	-0.045	-2.411×10^{-3}	-0.045*	0.266	0.114	0.079	0.183
							±0.023	±0.026	±0.022	±0.022				
							p =1.399×10^{-154}	p =0.110	p =0.912	p =0.042				
$shoot-lig.mass$	g	log	+	**		+	7.407***	1.532***	2.281***	1.151***	0.798	0.636	0.341	0.682
							±0.369	±0.322	±0.348	±0.212				
							p =1.787×10^{-89}	p =6.296×10^{-6}	p =3.788×10^{-10}	p =5.774×10^{-8}				
$shoot.m/height$	g cm$^{-\beta}$	log		***			-5.340***	-0.306***	-0.085		0.671	0.625	0.511	0.311
							±0.147	±0.035	±0.186					
							p =2.503×10^{-288}	p =1.096×10^{-16}	p =0.665					
SLA	cm^2 g^{-1}	log		***			4.519***	0.155***	0.319***		0.898	0.835	0.542	0.773
							±0.053	±0.013	±0.068					
							p =0	p =2.058×10^{-32}	p =7.537×10^{-6}					
$sPRI_{adax}$	unitless	exp		*		+	1.639***	0.011***	-0.011*		0.532	0.302	0.159	0.443
							±4.102×10^{-3}	±2.297×10^{-3}	±4.843×10^{-3}					
							p =0	p =1.303×10^{-5}	p =0.032					
$stomate.size$	μm^2	log		***		○	6.213***	0.052	-0.120		0.543	0.099	0.464	0.142
							±0.081	±0.031	±0.098					
							p =0	p =0.124	p =0.258					
$survival$	portion			+	+	+	0.925***	0.596***	0.054	0.360***	0.716	0.634	0.129	0.648
							±0.075	±0.071	±0.061	±0.063				
							p =1.331×10^{-34}	p =9.381×10^{-16}	p =0.404	p =1.344×10^{-8}				
TLP	MPa	log	**	***	+		-0.529***	0.097***	0.080*		0.748	0.25	0.329	0.609
							±0.045	±0.027	±0.035					

continued

Trait	Units	Trans	site	sp	phyl	attr	Intercept	site P/E_p	species P/E_p	Interact	R^2_{tot}	$R^2_{part\ fix}$	$R^2_{part\ rand}$	R^2_{fix}
							$p = 2.542\times10^{-31}$	$p = 6.244\times10^{-4}$	$p = 0.033$					
$V_{cmax}/mass$	μmol g^{-1} s^{-1}	sqrt	**	+			0.951***<br>±0.049<br>$p = 3.822\times10^{-83}$	0.058<br>±0.039<br>$p = 0.177$	0.088°<br>±0.042<br>$p = 0.052$		0.411	0.15	0.19	0.255
VLA	μm mm^{-2}						8.382×10^{3}***<br>±358.480<br>$p = 2.973\times10^{-21}$	-1.337×10^{3}***<br>±285.120<br>$p = 1.290\times10^{-4}$	-1.903×10^{3}***<br>±428.490<br>$p = 2.391\times10^{-4}$		0.631			0.631
WBI_{adax}	unitless		+	**			1.031***<br>$\pm3.071\times10^{-3}$<br>$p = 0$	-1.685×10^{-3}<br>$\pm1.574\times10^{-3}$<br>$p = 0.324$	-8.346×10^{-3}*<br>$\pm3.578\times10^{-3}$<br>$p = 0.032$		0.491	0.151	0.268	0.303
wood density	g cm^{-3}	sqrt		***			0.664***<br>±0.017<br>$p = 0$	-0.041***<br>$\pm3.375\times10^{-3}$<br>$p = 2.058\times10^{-32}$	-0.082***<br>±0.021<br>$p = 2.145\times10^{-4}$		0.899	0.823	0.651	0.711
A_{area}	μmol CO_2 m^{-2} s^{-1}			+		○	16.821***<br>±0.950<br>$p = 4.321\times10^{-70}$	3.665***<br>±0.695<br>$p = 4.853\times10^{-7}$	-1.761<br>±0.982<br>$p = 0.101$		0.634	0.34	0.14	0.57
A_{area}/g_s	μmol CO_2 mol H_2O^{-1}	log	+			+	4.057***<br>±0.054<br>$p = 0$	-0.443***<br>±0.051<br>$p = 1.063\times10^{-16}$	0.194***<br>±0.030<br>$p = 9.632\times10^{-10}$		0.929	0.652	0.288	0.896
A_{mass}	μmol CO_2 g^{-1} s^{-1}	sqrt		+	+	+	0.398***<br>±0.018<br>$p = 1.803\times10^{-111}$	0.067***<br>$\pm9.883\times10^{-3}$<br>$p = 6.839\times10^{-11}$	0.041**<br>±0.014<br>$p = 0.007$		0.728	0.447	0.134	0.675
bark.a/stem.a	mm^2 $mm^{-2\beta}$	log	*	***			-0.592***<br>±0.067<br>$p = 1.022\times10^{-18}$	0.027<br>±0.022<br>$p = 0.252$	-0.105<br>±0.081<br>$p = 0.235$		0.682	0.077	0.653	0.076
bark.dens	g cm^{-3}	log	***	*			-1.118***<br>±0.039<br>$p = 4.770\times10^{-182}$	-0.229***<br>±0.033<br>$p = 2.423\times10^{-11}$	-0.173***<br>±0.028<br>$p = 1.984\times10^{-9}$		0.924	0.528	0.324	0.887
basal.area	mm^2	log	+	**		+	7.450***<br>±0.307<br>$p = 3.963\times10^{-130}$	1.166***<br>±0.277<br>$p = 6.595\times10^{-5}$	1.972***<br>±0.269<br>$p = 2.601\times10^{-12}$	0.938***<br>±0.173<br>$p = 5.832\times10^{-8}$	0.81	0.645	0.346	0.693
c_i	μmol CO_2 mol air^{-1}	log	*				5.554***<br>±0.026<br>$p = 0$	0.196***<br>±0.024<br>$p = 2.886\times10^{-15}$	-0.078***<br>±0.020<br>$p = 1.803\times10^{-4}$		0.863	0.492	0.112	0.844
C_{mass}	mg g^{-1}	log		***			6.258***<br>$\pm9.171\times10^{-3}$<br>$p = 0$	1.286×10^{-3}<br>$\pm2.354\times10^{-3}$<br>$p = 0.608$	0.023°<br>±0.012<br>$p = 0.064$		0.653	0.106	0.519	0.276
CI_{adax}	unitless	exp		***		○	1.656***<br>±0.026<br>$p = 0$	0.020<br>±0.012<br>$p = 0.107$	0.060°<br>±0.031<br>$p = 0.070$		0.615	0.159	0.435	0.315

continued

Trait	Units	Trans	site	sp	phyl	attr	Intercept	site P/E_p	species P/E_p	Interact	R^2_{tot}	$R^2_{part\ fix}$	$R^2_{part\ rand}$	R^2_{fix}
conduit.dens	mm^{-2}	log	*	*	+		4.182***<br>±0.127<br>$p = 1.944\times10^{-236}$	-0.081<br>±0.081<br>$p = 0.348$	-0.561***<br>±0.102<br>$p = 1.627\times10^{-7}$		0.667	0.31	0.227	0.553
cuticle.thick	μm	sqrt				+	2.243***<br>±0.138<br>$p = 1.901\times10^{-59}$	-0.018<br>±0.128<br>$p = 0.896$	-0.416***<br>±0.110<br>$p = 3.323\times10^{-4}$		0.283	0.27	0.075	0.177
D_H	μm	log	*	*	+		3.747***<br>±0.078<br>$p = 0$	0.077<br>±0.049<br>$p = 0.144$	0.333***<br>±0.060<br>$p = 1.265\times10^{-7}$		0.642	0.339	0.199	0.54
$\delta^{13}C$	‰	log	+	+			-3.372***<br>$\pm 9.049\times10^{-3}$<br>$p = 0$	-0.067***<br>$\pm 6.403\times10^{-3}$<br>$p = 2.203\times10^{-24}$	-1.993×10^{-3}<br>$\pm 9.410\times10^{-3}$<br>$p = 0.848$		0.86	0.332	0.09	0.846
dx/dy	$\mu m\ \mu m^{-1}$	log		*			0.414***<br>±0.057<br>$p = 6.156\times10^{-13}$	0.140***<br>±0.028<br>$p = 1.982\times10^{-6}$	0.328***<br>±0.072<br>$p = 1.341\times10^{-5}$		0.712	0.597	0.136	0.665
epid.cell.size	μm^2	log		*		○	5.915***<br>±0.089<br>$p = 0$	0.107°<br>±0.053<br>$p = 0.062$	-0.139<br>±0.102<br>$p = 0.209$		0.351	0.127	0.171	0.21
g_{min}	mmol $H_2O\ m^{-2}\ s^{-1}$	log				**	2.332***<br>±0.187<br>$p = 7.754\times10^{-36}$	0.989***<br>±0.174<br>$p = 6.447\times10^{-8}$	-0.614***<br>±0.142<br>$p = 4.220\times10^{-5}$		0.743	0.544	0.22	0.665
g_s	mol $H_2O\ m^{-2}\ s^{-1}$	sqrt	+	+		+	0.553***<br>±0.030<br>$p = 7.585\times10^{-76}$	0.165***<br>±0.026<br>$p = 1.326\times10^{-9}$	-0.077***<br>±0.021<br>$p = 5.243\times10^{-4}$		0.87	0.398	0.244	0.825
height	cm	sqrt	+	**	+	+	18.539***<br>±2.221<br>$p = 7.085\times10^{-17}$	5.489***<br>±0.851<br>$p = 7.018\times10^{-10}$	6.670***<br>±0.912<br>$p = 2.845\times10^{-12}$	3.252***<br>±0.581<br>$p = 2.123\times10^{-8}$	0.834	0.697	0.279	0.755
$J_{max}/mass$	$\mu mol\ g^{-1}\ s^{-1}$		**	**			1.563***<br>±0.185<br>$p = 3.570\times10^{-17}$	0.227<br>±0.128<br>$p = 0.103$	0.162<br>±0.177<br>$p = 0.384$		0.417	0.091	0.287	0.13
k_{leaf}	mmol $s^{-1}\ m^{-2}\ MPa^{-1}$	log					2.358***<br>±0.149<br>$p = 2.210\times10^{-16}$	0.598***<br>±0.119<br>$p = 5.236\times10^{-5}$	-0.483*<br>±0.178<br>$p = 0.019$		0.476			0.476
K_s	g $s^{-1}\ MPa^{-1}\ mm^{-1}$	log		*		○	0.550***<br>±0.118<br>$p = 2.952\times10^{-6}$	0.376***<br>±0.076<br>$p = 2.249\times10^{-6}$	0.503***<br>±0.130<br>$p = 2.439\times10^{-4}$		0.735	0.396	0.143	0.689
$K_{stem.theo}/xyl.a$	g $s^{-1}\ MPa^{-1}\ mm^{-1}$	log	+	+	+		1.635***<br>±0.200<br>$p = 3.226\times10^{-16}$	0.223<br>±0.129<br>$p = 0.110$	0.755***<br>±0.178<br>$p = 5.629\times10^{-5}$		0.506	0.288	0.1	0.445
$K_{stem}/lvs.a$	g mm $s^{-1}\ MPa^{-1}\ mm^{-2}$	log	+	+			5.917***	0.289*	0.532***		0.558	0.311	0.067	0.523

continued

Trait	Units	Trans	site	sp	phyl	attr	Intercept	site P/E_p	species P/E_p	Interact	R^2_{tot}	$R^2_{part\ fix}$	$R^2_{part\ rand}$	R^2_{fix}
							±0.144	±0.110	±0.142					
							$p=0$	$p=0.015$	$p=3.857\times10^{-4}$					
leaf.size.area	cm^2	sqrt		***		+	3.945***	0.530***	0.452		0.731	0.331	0.606	0.31
							±0.362	±0.099	±0.453					
							$p=1.329\times10^{-27}$	$p=3.813\times10^{-7}$	$p=0.348$					
leaf.thick	μm			*			284.120***	-24.957**	-98.475***		0.712	0.493	0.167	0.651
							±16.062	±7.569	±20.025					
							$p=5.146\times10^{-70}$	$p=0.002$	$p=2.901\times10^{-6}$					
leaves.area	cm^2	log		**		***	10.902***	1.542***	2.362***	1.124***	0.814	0.657	0.345	0.713
							±0.326	±0.285	±0.338	±0.212				
							$p=2.428\times10^{-245}$	$p=2.578\times10^{-7}$	$p=2.423\times10^{-11}$	$p=1.146\times10^{-7}$				
lvs.a/basal.a	cm^2 mm$^{-2\beta}$	sqrt		***	+	+	5.076***	0.304***	0.759**		0.687	0.374	0.419	0.439
							±0.235	±0.070	±0.220					
							$p=9.032\times10^{-104}$	$p=4.570\times10^{-5}$	$p=0.001$					
lvs.a/stem.m	cm^2 g$^{-\beta}$	sqrt		***		*	14.393***	1.742***	1.627**	0.854**	0.744	0.502	0.353	0.603
							±0.431	±0.307	±0.515	±0.268				
							$p=3.597\times10^{-244}$	$p=6.447\times10^{-8}$	$p=0.003$	$p=0.001$				
lvs.m/root.m	g g$^{-\beta}$	log					1.293***	0.388**	0.090	0.354**	0.316			0.316
							±0.074	±0.105	±0.093	±0.111				
							$p=2.664\times10^{-17}$	$p=0.002$	$p=0.374$	$p=0.003$				
lvs.m/shoot.m	g g$^{-\beta}$	exp		***			2.125***	0.162***	-0.088	0.155***	0.607	0.352	0.355	0.368
							±0.067	±0.041	±0.085	±0.043				
							$p=2.021\times10^{-223}$	$p=2.063\times10^{-4}$	$p=0.336$	$p=2.953\times10^{-4}$				
n.trunks	count	log		***	+	+	0.614*	-0.098	-0.635*		0.61	0.137	0.478	0.222
							±0.274	±0.071	±0.278					
							$p=0.025$	$p=0.206$	$p=0.035$					
N_{area}	mg cm^{-2}	sqrt		+		**	0.449***	-0.049***	-0.030***		0.866	0.533	0.208	0.829
							$\pm8.198\times10^{-3}$	$\pm6.863\times10^{-3}$	$\pm7.188\times10^{-3}$					
							$p=0$	$p=8.164\times10^{-12}$	$p=6.595\times10^{-5}$					
N_{mass}	mg g^{-1}	log		***		*	2.914***	-0.044	0.192*		0.645	0.167	0.475	0.314
							±0.066	±0.029	±0.079					
							$p=0$	$p=0.165$	$p=0.025$					
$NDVI_{adax}$	unitless	exp		***		+	2.260***	0.033°	0.161*		0.734	0.195	0.579	0.365
							±0.057	±0.016	±0.071					
							$p=0$	$p=0.054$	$p=0.037$					
pore.length	μm^2	log		***	+		2.396***	-0.023	-0.138**		0.495	0.214	0.336	0.196
							±0.049	±0.015	±0.040					
							$p=0$	$p=0.165$	$p=0.001$					
ψ_{md}	MPa	log	***	*			-0.420***	0.286**	0.092		0.733	0.164	0.393	0.555
							±0.113	±0.095	±0.081					

continued

Trait	Units	Trans	site	sp	phyl	attr	Intercept	site P/E_p	species P/E_p	Interact	R^2_{tot}	$R^2_{part\ fix}$	$R^2_{part\ rand}$	R^2_{fix}
							$p=2.054\times10^{-4}$	$p=0.005$	$p=0.294$					
ψ_{pd}	MPa	log	*	+		+	0.517*<br>±0.212<br>$p=0.015$	0.576**<br>±0.190<br>$p=0.004$	0.124<br>±0.124<br>$p=0.348$		0.768	0.159	0.412	0.596
$refl_{adax}$	unitless	log		***		+	-2.819***<br>±0.138<br>$p=2.291\times10^{-92}$	-0.067<br>±0.037<br>$p=0.101$	−0.322°<br>±0.173<br>$p=0.088$		0.701	0.149	0.578	0.29
$refl_{adax/abax}$	unitless	log		***	*		-0.274***<br>±0.080<br>$p=5.629\times10^{-4}$	−0.045°<br>±0.021<br>$p=0.052$	-0.037<br>±0.055<br>$p=0.528$		0.558	0.126	0.445	0.14
RGR.height	cm cm^{-1} day^{-1}			+		+	2.583***<br>±0.122<br>$p=6.230\times10^{-99}$	1.313***<br>±0.142<br>$p=7.443\times10^{-19}$	0.886***<br>±0.134<br>$p=2.787\times10^{-10}$	0.827***<br>±0.131<br>$p=3.118\times10^{-10}$	0.841	0.714	0.078	0.824
RGR.shoot	g g^{-1} day^{-1}						6.009***<br>±0.230<br>$p=3.208\times10^{-22}$	2.539***<br>±0.326<br>$p=5.621\times10^{-8}$	2.206***<br>±0.291<br>$p=8.068\times10^{-8}$	2.141***<br>±0.345<br>$p=7.765\times10^{-7}$	0.771			0.771
root.m/shoot.m	g $g^{-\beta}$	sqrt	○	+			0.604***<br>±0.023<br>$p=1.399\times10^{-154}$	-0.045<br>±0.026<br>$p=0.110$	-2.411×10^{-3}<br>±0.022<br>$p=0.912$	-0.045*<br>±0.022<br>$p=0.042$	0.266	0.114	0.079	0.183
$shoot-lig.mass$	g	log	+	**		+	7.407***<br>±0.369<br>$p=1.787\times10^{-89}$	1.532***<br>±0.322<br>$p=6.296\times10^{-6}$	2.281***<br>±0.348<br>$p=3.788\times10^{-10}$	1.151***<br>±0.212<br>$p=5.774\times10^{-8}$	0.798	0.636	0.341	0.682
shoot.m/height	g $cm^{-\beta}$	log		***			-5.340***<br>±0.147<br>$p=2.503\times10^{-288}$	-0.306***<br>±0.035<br>$p=1.096\times10^{-16}$	-0.085<br>±0.186<br>$p=0.665$		0.671	0.625	0.511	0.311
SLA	cm^2 g^{-1}	log		***			4.519***<br>±0.053<br>$p=0$	0.155***<br>±0.013<br>$p=2.058\times10^{-32}$	0.319***<br>±0.068<br>$p=7.537\times10^{-6}$		0.898	0.835	0.542	0.773
$sPRI_{adax}$	unitless	exp		*		+	1.639***<br>$\pm4.102\times10^{-3}$<br>$p=0$	0.011***<br>$\pm2.297\times10^{-3}$<br>$p=1.303\times10^{-5}$	-0.011*<br>$\pm4.843\times10^{-3}$<br>$p=0.032$		0.532	0.302	0.159	0.443
stomate.size	μm^2	log		***		○	6.213***<br>±0.081<br>$p=0$	0.052<br>±0.031<br>$p=0.124$	-0.120<br>±0.098<br>$p=0.258$		0.543	0.099	0.464	0.142
survival	portion			+	+	+	0.925***<br>±0.075<br>$p=1.331\times10^{-34}$	0.596***<br>±0.071<br>$p=9.381\times10^{-16}$	0.054<br>±0.061<br>$p=0.404$	0.360***<br>±0.063<br>$p=1.344\times10^{-8}$	0.716	0.634	0.129	0.648
TLP	MPa	log	**	***	+		-0.529***<br>±0.045<br>$p=2.542\times10^{-31}$	0.097***<br>±0.027<br>$p=6.244\times10^{-4}$	0.080*<br>±0.035<br>$p=0.033$		0.748	0.25	0.329	0.609

continued

Trait	Units	Trans	site	sp	phyl	attr	Intercept	site P/E_p	species P/E_p	Interact	R^2_{tot}	$R^2_{part\ fix}$	$R^2_{part\ rand}$	R^2_{fix}
$V_{cmax}/mass$	μmol g^{-1} s^{-1}	sqrt	**	+			0.951***<br>$\pm$0.049<br>p =3.822$\times 10^{-83}$	0.058<br>$\pm$0.039<br>p =0.177	0.088°<br>$\pm$0.042<br>p =0.052		0.411	0.15	0.19	0.255
VLA	μm mm^{-2}						8.382$\times 10^{3}$***<br>$\pm$358.480<br>p =2.973$\times 10^{-21}$	-1.337$\times 10^{3}$***<br>$\pm$285.120<br>p =1.290$\times 10^{-4}$	-1.903$\times 10^{3}$***<br>$\pm$428.490<br>p =2.391$\times 10^{-4}$		0.631			0.631
WBI_{adax}	unitless		+	**			1.031***<br>$\pm$3.071$\times 10^{-3}$<br>p =0	-1.685$\times 10^{-3}$<br>$\pm$1.574$\times 10^{-3}$<br>p =0.324	-8.346$\times 10^{-3}$*<br>$\pm$3.578$\times 10^{-3}$<br>p =0.032		0.491	0.151	0.268	0.303
$wood\ density$	g cm^{-3}	sqrt		***			0.664***<br>$\pm$0.017<br>p =0	-0.041***<br>$\pm$3.375$\times 10^{-3}$<br>p =2.058$\times 10^{-32}$	-0.082***<br>$\pm$0.021<br>p =2.145$\times 10^{-4}$		0.899	0.823	0.651	0.711

Table S4: Results of non-phylogenetically structured regression of traits, transformed as indicated for normalization. Fixed effects were log-transformed site P/E_p, species P/E_p and their interaction. Random effect columns indicate. Fixed effect columns give coefficients with significance symbolized as ***= $p < 0.001$, **= $p < 0.01$, *= $p < 0.05$, and $\circ$ = $p < 0.1$. Standard error and exact p values follow coefficients. The p values for species P/E_p and site P/E_p were adjusted with Yekutieli-Benjamini-Hochberg procedure. All fixed effects except interaction were always kept. Interaction was kept when $p < 0.05$. Unit exponents β control for allometric scaling of trait ratios (see Source Data).

Trait	Units	Trans	Intercept	site P/E_p	species P/E_p	Interact	R^2
A_{area}	μmol CO_2 m^{-2} s^{-1}		16.662***	3.604***	-2.142*		0.574
			$\pm$0.713	$\pm$0.567	$\pm$0.852		
			p =2.999×10^{-21}	p =2.255×10^{-6}	p =0.026		
A_{area}/g_s	μmol CO_2 mol H_2O^{-1}	log	4.061***	-0.469***	0.207***		0.9
			$\pm$0.035	$\pm$0.028	$\pm$0.042		
			p =2.679×10^{-42}	p =5.842×10^{-15}	p =9.958×10^{-5}		
A_{mass}	μmol CO_2 g^{-1} s^{-1}	sqrt	0.391***	0.065***	0.030*		0.686
			$\pm$0.011	$\pm$8.903×10^{-3}	$\pm$0.013		
			p =1.787×10^{-26}	p =2.649×10^{-7}	p =0.049		
$bark.a/stem.a$	mm^2 $mm^{-2\beta}$	log	-0.579***	0.015	-0.084		0.085
			$\pm$0.042	$\pm$0.033	$\pm$0.050		
			p =6.726×10^{-15}	p =0.700	p =0.129		
$bark.dens$	g cm^{-3}	log	-1.115***	-0.240***	-0.165***		0.888
			$\pm$0.023	$\pm$0.019	$\pm$0.028		
			p =1.328×10^{-30}	p =1.707×10^{-12}	p =6.504×10^{-6}		
$basal.area$	mm^2	log	7.420***	0.829*	1.943***	0.677*	0.709
			$\pm$0.204	$\pm$0.290	$\pm$0.258	$\pm$0.306	
			p =2.158×10^{-26}	p =0.012	p =1.737×10^{-7}	p =0.035	
c_i	μmol CO_2 mol air^{-1}	log	5.551***	0.205***	-0.086**		0.846
			$\pm$0.020	$\pm$0.016	$\pm$0.024		
			p =2.270×10^{-54}	p =1.707×10^{-12}	p =2.135×10^{-3}		
C_{mass}	mg g^{-1}	log	6.259***	3.462×10^{-4}	0.025**		0.279
			$\pm$6.195×10^{-3}	$\pm$4.927×10^{-3}	$\pm$7.405×10^{-3}		
			p =1.350×10^{-71}	p =0.944	p =4.025×10^{-3}		
CI_{adax}	unitless	exp	1.660***	0.017	0.068**		0.319
			$\pm$0.017	$\pm$0.014	$\pm$0.021		
			p =5.978×10^{-40}	p =0.276	p =4.805×10^{-3}		
$conduit.dens$	mm^{-2}	log	4.199***	-0.035	-0.531***		0.57
			$\pm$0.073	$\pm$0.058	$\pm$0.087		
			p =4.072×10^{-33}	p =0.595	p =4.000×10^{-6}		
$cuticle.thick$	μm	sqrt	2.297***	-0.121	-0.300*		0.225

continued

Trait	Units	Trans	Intercept	site P/E_p	species P/E_p	Interact	R^2
			±0.109	±0.087	±0.131		
			$p=6.561\times10^{-20}$	$p=0.208$	$p=0.041$		
D_H	μm	log	3.734***	0.057	0.308***		0.552
			±0.047	±0.037	±0.056		
			$p=1.639\times10^{-37}$	$p=0.171$	$p=1.790\times10^{-5}$		
$\delta^{13}C$	‰	log	-3.372***	-0.069***	-1.653×10^{-3}		0.846
			$\pm6.782\times10^{-3}$	$\pm5.394\times10^{-3}$	$\pm8.106\times10^{-3}$		
			$p=4.754\times10^{-62}$	$p=2.055\times10^{-12}$	$p=0.864$		
dx/dy	μm μm^{-1}	log	0.419***	0.127**	0.336***		0.667
			±0.046	±0.036	±0.055		
			$p=2.521\times10^{-10}$	$p=3.004\times10^{-3}$	$p=3.868\times10^{-6}$		
$epid.cell.size$	μm^2	log	5.904***	0.129*	$-0.156°$		0.217
			±0.065	±0.052	±0.078		
			$p=3.262\times10^{-39}$	$p=0.026$	$p=0.072$		
g_{min}	mmol H_2O m^{-2} s^{-1}	log	2.386***	0.969***	-0.497*		0.67
			±0.155	±0.123	±0.185		
			$p=4.464\times10^{-16}$	$p=8.569\times10^{-8}$	$p=0.018$		
g_s	mol H_2O m^{-2} s^{-1}	sqrt	0.548***	0.175***	-0.089***		0.828
			±0.018	±0.014	±0.022		
			$p=1.459\times10^{-24}$	$p=5.522\times10^{-12}$	$p=6.857\times10^{-4}$		
$height$	cm	sqrt	18.044***	4.401***	6.423***	2.612*	0.769
			±0.671	±0.954	±0.849	±1.008	
			$p=1.463\times10^{-22}$	$p=2.026\times10^{-4}$	$p=1.737\times10^{-7}$	$p=0.015$	
$J_{max}/mass$	μmol g^{-1} s^{-1}		1.608***	0.122	0.241°		0.182
			±0.109	±0.086	±0.130		
			$p=1.330\times10^{-15}$	$p=0.201$	$p=0.097$		
k_{leaf}	mmol s^{-1} m^{-2} MPa^{-1}	log	2.358***	0.598***	-0.483*		0.476
			±0.149	±0.119	±0.178		
			$p=2.210\times10^{-16}$	$p=6.587\times10^{-5}$	$p=0.018$		
K_s	g s^{-1} MPa^{-1} mm^{-1}	log	0.563***	0.353***	0.524***		0.691
			±0.084	±0.067	±0.101		
			$p=1.898\times10^{-7}$	$p=3.743\times10^{-5}$	$p=4.406\times10^{-5}$		
$K_{stem.theo}/xyl.a$	g s^{-1} MPa^{-1} mm^{-1}	log	1.607***	0.192	0.700***		0.451
			±0.138	±0.110	±0.165		
			$p=7.152\times10^{-13}$	$p=0.117$	$p=4.705\times10^{-4}$		
$K_{stem}/lvs.a$	g mm s^{-1} MPa^{-1} mm^{-2}	log	5.937***	0.251*	0.569***		0.527
			±0.109	±0.087	±0.130		
			$p=2.290\times10^{-32}$	$p=0.011$	$p=3.495\times10^{-4}$		
$leaf.size.area$	cm^2	sqrt	3.882***	0.608**	0.349		0.316
			±0.236	±0.188	±0.283		

continued

Trait	Units	Trans	Intercept	site P/E_p	species P/E_p	Interact	R^2
			$p = 7.571 \times 10^{-17}$	$p = 5.177 \times 10^{-3}$	$p = 0.266$		
leaf.thick	μm		281.640***	-20.413°	-102.820***		0.654
			±12.687	±10.090	±15.165		
			$p = 1.363 \times 10^{-20}$	$p = 0.072$	$p = 7.597 \times 10^{-7}$		
leaves.area	cm^2	log	10.828***	1.361**	2.245***	0.907*	0.716
			±0.253	±0.359	±0.320	±0.380	
			$p = 1.792 \times 10^{-28}$	$p = 1.570 \times 10^{-3}$	$p = 5.451 \times 10^{-7}$	$p = 0.023$	
lvs.a/basal.a	cm^2 $mm^{-2\beta}$	sqrt	4.986***	0.283*	0.599**		0.461
			±0.134	±0.106	±0.160		
			$p = 2.575 \times 10^{-27}$	$p = 0.019$	$p = 1.646 \times 10^{-3}$		
lvs.a/stem.m	cm^2 $g^{-\beta}$	sqrt	14.394***	1.886***	1.637***	0.998*	0.605
			±0.322	±0.458	±0.408	±0.484	
			$p = 5.212 \times 10^{-29}$	$p = 6.857 \times 10^{-4}$	$p = 8.881 \times 10^{-4}$	$p = 0.048$	
lvs.m/root.m	g $g^{-\beta}$	log	1.293***	0.388**	0.090	0.354**	0.316
			±0.074	±0.105	±0.093	±0.111	
			$p = 2.664 \times 10^{-17}$	$p = 1.882 \times 10^{-3}$	$p = 0.394$	$p = 3.287 \times 10^{-3}$	
lvs.m/shoot.m	g $g^{-\beta}$	exp	2.124***	0.225**	-0.089	0.201*	0.391
			±0.049	±0.070	±0.062	±0.074	
			$p = 1.499 \times 10^{-28}$	$p = 5.502 \times 10^{-3}$	$p = 0.201$	$p = 0.011$	
n.trunks	count	log	0.655***	-0.013	-0.552**		0.254
			±0.147	±0.117	±0.175		
			$p = 9.666 \times 10^{-5}$	$p = 0.921$	$p = 6.240 \times 10^{-3}$		
N_{area}	mg cm^{-2}	sqrt	0.447***	-0.050***	-0.034***		0.831
			$\pm 6.183 \times 10^{-3}$	$\pm 4.917 \times 10^{-3}$	$\pm 7.390 \times 10^{-3}$		
			$p = 3.952 \times 10^{-36}$	$p = 3.140 \times 10^{-10}$	$p = 2.329 \times 10^{-4}$		
N_{mass}	mg g^{-1}	log	2.918***	-0.061	0.193**		0.324
			±0.043	±0.034	±0.052		
			$p = 3.437 \times 10^{-35}$	$p = 0.112$	$p = 1.729 \times 10^{-3}$		
$NDVI_{adax}$	unitless	exp	2.266***	0.026	0.168**		0.367
			±0.036	±0.029	±0.043		
			$p = 3.898 \times 10^{-34}$	$p = 0.421$	$p = 1.270 \times 10^{-3}$		
pore.length	μm^2	log	2.415***	-0.012	-0.103*		0.239
			±0.030	±0.024	±0.035		
			$p = 9.674 \times 10^{-38}$	$p = 0.643$	$p = 0.011$		
ψ_{md}	MPa	log	-0.430***	0.314***	0.066		0.56
			±0.067	±0.053	±0.080		
			$p = 3.686 \times 10^{-7}$	$p = 6.504 \times 10^{-6}$	$p = 0.464$		
ψ_{pd}	MPa	log	0.484***	0.647***	0.051		0.605
			±0.122	±0.097	±0.146		
			$p = 4.100 \times 10^{-4}$	$p = 1.025 \times 10^{-6}$	$p = 0.768$		

continued

Trait	Units	Trans	Intercept	site P/E_p	species P/E_p	Interact	R^2
$refl_{adax}$	unitless	log	-2.836***<br>±0.087<br>p =1.614×10^{-25}	-0.052<br>±0.069<br>p =0.511	-0.340**<br>±0.104<br>p =4.897×10^{-3}		0.292
$refl_{adax/abax}$	unitless	log	-0.311***<br>±0.045<br>p =1.056×10^{-7}	-0.053<br>±0.036<br>p =0.185	−0.110°<br>±0.054<br>p =0.072		0.203
$RGR.height$	cm cm^{-1} day^{-1}		2.632***<br>±0.108<br>p =2.423×10^{-21}	1.392***<br>±0.153<br>p =6.299×10^{-9}	0.973***<br>±0.137<br>p =4.482×10^{-7}	0.933***<br>±0.162<br>p =2.747×10^{-6}	0.828
$RGR.shoot$	g g^{-1} day^{-1}		6.009***<br>±0.230<br>p =3.208×10^{-22}	2.539***<br>±0.326<br>p =1.180×10^{-7}	2.206***<br>±0.291<br>p =1.737×10^{-7}	2.141***<br>±0.345<br>p =7.765×10^{-7}	0.771
$root.m/shoot.m$	g g$^{-\beta}$	sqrt	0.602***<br>±0.018<br>p =2.458×10^{-25}	-0.066*<br>±0.026<br>p =0.023	-3.697×10^{-3}<br>±0.023<br>p =0.889	-0.063*<br>±0.027<br>p =0.027	0.203
$shoot-lig.mass$	g	log	7.364***<br>±0.252<br>p =1.274×10^{-23}	1.185**<br>±0.358<br>p =4.570×10^{-3}	2.229***<br>±0.319<br>p =5.451×10^{-7}	0.883*<br>±0.378<br>p =0.026	0.693
$shoot.m/height$	g cm$^{-\beta}$	log	-5.371***<br>±0.094<br>p =6.070×10^{-33}	-0.254**<br>±0.075<br>p =3.866×10^{-3}	-0.128<br>±0.113<br>p =0.305		0.327
SLA	cm^2 g^{-1}	log	4.526***<br>±0.035<br>p =5.988×10^{-44}	0.138***<br>±0.028<br>p =7.801×10^{-5}	0.328***<br>±0.042<br>p =8.569×10^{-8}		0.777
$sPRI_{adax}$	unitless	exp	1.638***<br>±3.061×10^{-3}<br>p =4.819×10^{-63}	0.011***<br>±2.434×10^{-3}<br>p =3.901×10^{-4}	-0.012**<br>±3.659×10^{-3}<br>p =4.439×10^{-3}		0.444
$stomate.size$	μm^2	log	6.218***<br>±0.051<br>p =4.979×10^{-43}	0.069<br>±0.041<br>p =0.129	−0.117°<br>±0.061<br>p =0.089		0.147
$survival$	portion		0.905***<br>±0.055<br>p =1.152×10^{-16}	0.541***<br>±0.078<br>p =5.451×10^{-7}	0.017<br>±0.069<br>p =0.839	0.345***<br>±0.082<br>p =2.141×10^{-4}	0.674
TLP	MPa	log	-0.522***<br>±0.025<br>p =7.144×10^{-20}	0.112***<br>±0.020<br>p =1.311×10^{-5}	0.093**<br>±0.030<br>p =6.335×10^{-3}		0.625
$V_{cmax}/mass$	μmol g^{-1} s^{-1}	sqrt	0.959***<br>±0.032<br>p =2.621×10^{-24}	0.039<br>±0.026<br>p =0.180	0.103*<br>±0.039<br>p =0.019		0.272
VLA	μm mm^{-2}		8.382×10^3***	-1.337×10^3***	-1.903×10^3***		0.631

continued

Trait	Units	Trans	Intercept	site P/E_p	species P/E_p	Interact	R^2
			±358.480	±285.120	±428.490		
			$p=2.973\times10^{-21}$	$p=1.585\times10^{-4}$	$p=2.958\times10^{-4}$		
WBI_{adax}	unitless		1.031***	-1.212×10^{-3}	-8.784×10^{-3}**		0.305
			$\pm2.167\times10^{-3}$	$\pm1.723\times10^{-3}$	$\pm2.590\times10^{-3}$		
			$p=1.859\times10^{-61}$	$p=0.532$	$p=3.866\times10^{-3}$		
wood density	g cm^{-3}	sqrt	0.664***	-0.042***	-0.082***		0.711
			±0.011	$\pm8.885\times10^{-3}$	±0.013		
			$p=1.611\times10^{-33}$	$p=1.272\times10^{-4}$	$p=3.938\times10^{-6}$		
A_{area}	μmol CO_2 m^{-2} s^{-1}		16.662***	3.604***	-2.142*		0.574
			±0.713	±0.567	±0.852		
			$p=2.999\times10^{-21}$	$p=2.255\times10^{-6}$	$p=0.026$		
A_{area}/g_s	μmol CO_2 mol H_2O^{-1}	log	4.061***	-0.469***	0.207***		0.9
			±0.035	±0.028	±0.042		
			$p=2.679\times10^{-42}$	$p=5.842\times10^{-15}$	$p=9.958\times10^{-5}$		
A_{mass}	μmol CO_2 g^{-1} s^{-1}	sqrt	0.391***	0.065***	0.030*		0.686
			±0.011	$\pm8.903\times10^{-3}$	±0.013		
			$p=1.787\times10^{-26}$	$p=2.649\times10^{-7}$	$p=0.049$		
bark.a/stem.a	mm^2 $mm^{-2\beta}$	log	-0.579***	0.015	-0.084		0.085
			±0.042	±0.033	±0.050		
			$p=6.726\times10^{-15}$	$p=0.700$	$p=0.129$		
bark.dens	g cm^{-3}	log	-1.115***	-0.240***	-0.165***		0.888
			±0.023	±0.019	±0.028		
			$p=1.328\times10^{-30}$	$p=1.707\times10^{-12}$	$p=6.504\times10^{-6}$		
basal.area	mm^2	log	7.420***	0.829*	1.943***	0.677*	0.709
			±0.204	±0.290	±0.258	±0.306	
			$p=2.158\times10^{-26}$	$p=0.012$	$p=1.737\times10^{-7}$	$p=0.035$	
c_i	μmol CO_2 mol air^{-1}	log	5.551***	0.205***	-0.086**		0.846
			±0.020	±0.016	±0.024		
			$p=2.270\times10^{-54}$	$p=1.707\times10^{-12}$	$p=2.135\times10^{-3}$		
C_{mass}	mg g^{-1}	log	6.259***	3.462×10^{-4}	0.025**		0.279
			$\pm6.195\times10^{-3}$	$\pm4.927\times10^{-3}$	$\pm7.405\times10^{-3}$		
			$p=1.350\times10^{-71}$	$p=0.944$	$p=4.025\times10^{-3}$		
CI_{adax}	unitless	exp	1.660***	0.017	0.068**		0.319
			±0.017	±0.014	±0.021		
			$p=5.978\times10^{-40}$	$p=0.276$	$p=4.805\times10^{-3}$		
conduit.dens	mm^{-2}	log	4.199***	-0.035	-0.531***		0.57
			±0.073	±0.058	±0.087		
			$p=4.072\times10^{-33}$	$p=0.595$	$p=4.000\times10^{-6}$		
cuticle.thick	μm	sqrt	2.297***	-0.121	-0.300*		0.225
			±0.109	±0.087	±0.131		

continued

Trait	Units	Trans	Intercept	site P/E_p	species P/E_p	Interact	R^2
			$p = 6.561\times10^{-20}$	$p = 0.208$	$p = 0.041$		
D_H	μm	log	3.734***	0.057	0.308***		0.552
			±0.047	±0.037	±0.056		
			$p = 1.639\times10^{-37}$	$p = 0.171$	$p = 1.790\times10^{-5}$		
$\delta^{13}C$	‰	log	-3.372***	-0.069***	-1.653×10^{-3}		0.846
			$\pm6.782\times10^{-3}$	$\pm5.394\times10^{-3}$	$\pm8.106\times10^{-3}$		
			$p = 4.754\times10^{-62}$	$p = 2.055\times10^{-12}$	$p = 0.864$		
dx/dy	μm μm^{-1}	log	0.419***	0.127**	0.336***		0.667
			±0.046	±0.036	±0.055		
			$p = 2.521\times10^{-10}$	$p = 3.004\times10^{-3}$	$p = 3.868\times10^{-6}$		
$epid.cell.size$	μm^2	log	5.904***	0.129*	−0.156°		0.217
			±0.065	±0.052	±0.078		
			$p = 3.262\times10^{-39}$	$p = 0.026$	$p = 0.072$		
g_{min}	mmol H_2O m^{-2} s^{-1}	log	2.386***	0.969***	-0.497*		0.67
			±0.155	±0.123	±0.185		
			$p = 4.464\times10^{-16}$	$p = 8.569\times10^{-8}$	$p = 0.018$		
g_s	mol H_2O m^{-2} s^{-1}	sqrt	0.548***	0.175***	-0.089***		0.828
			±0.018	±0.014	±0.022		
			$p = 1.459\times10^{-24}$	$p = 5.522\times10^{-12}$	$p = 6.857\times10^{-4}$		
$height$	cm	sqrt	18.044***	4.401***	6.423***	2.612*	0.769
			±0.671	±0.954	±0.849	±1.008	
			$p = 1.463\times10^{-22}$	$p = 2.026\times10^{-4}$	$p = 1.737\times10^{-7}$	$p = 0.015$	
$J_{max}/mass$	μmol g^{-1} s^{-1}		1.608***	0.122	0.241°		0.182
			±0.109	±0.086	±0.130		
			$p = 1.330\times10^{-15}$	$p = 0.201$	$p = 0.097$		
k_{leaf}	mmol s^{-1} m^{-2} MPa^{-1}	log	2.358***	0.598***	-0.483*		0.476
			±0.149	±0.119	±0.178		
			$p = 2.210\times10^{-16}$	$p = 6.587\times10^{-5}$	$p = 0.018$		
K_s	g s^{-1} MPa^{-1} mm^{-1}	log	0.563***	0.353***	0.524***		0.691
			±0.084	±0.067	±0.101		
			$p = 1.898\times10^{-7}$	$p = 3.743\times10^{-5}$	$p = 4.406\times10^{-5}$		
$K_{stem.theo}/xyl.a$	g s^{-1} MPa^{-1} mm^{-1}	log	1.607***	0.192	0.700***		0.451
			±0.138	±0.110	±0.165		
			$p = 7.152\times10^{-13}$	$p = 0.117$	$p = 4.705\times10^{-4}$		
$K_{stem}/lvs.a$	g mm s^{-1} MPa^{-1} mm^{-2}	log	5.937***	0.251*	0.569***		0.527
			±0.109	±0.087	±0.130		
			$p = 2.290\times10^{-32}$	$p = 0.011$	$p = 3.495\times10^{-4}$		
$leaf.size.area$	cm^2	sqrt	3.882***	0.608**	0.349		0.316
			±0.236	±0.188	±0.283		
			$p = 7.571\times10^{-17}$	$p = 5.177\times10^{-3}$	$p = 0.266$		

continued

Trait	Units	Trans	Intercept	site P/E_p	species P/E_p	Interact	R^2
leaf.thick	μm		281.640***	$-20.413°$	-102.820***		0.654
			$\pm$12.687	$\pm$10.090	$\pm$15.165		
			$p=1.363\times10^{-20}$	$p=0.072$	$p=7.597\times10^{-7}$		
leaves.area	cm^2	log	10.828***	1.361**	2.245***	0.907*	0.716
			$\pm$0.253	$\pm$0.359	$\pm$0.320	$\pm$0.380	
			$p=1.792\times10^{-28}$	$p=1.570\times10^{-3}$	$p=5.451\times10^{-7}$	$p=0.023$	
lvs.a/basal.a	cm^2 mm$^{-2\beta}$	sqrt	4.986***	0.283*	0.599**		0.461
			$\pm$0.134	$\pm$0.106	$\pm$0.160		
			$p=2.575\times10^{-27}$	$p=0.019$	$p=1.646\times10^{-3}$		
lvs.a/stem.m	cm^2 g$^{-\beta}$	sqrt	14.394***	1.886***	1.637***	0.998*	0.605
			$\pm$0.322	$\pm$0.458	$\pm$0.408	$\pm$0.484	
			$p=5.212\times10^{-29}$	$p=6.857\times10^{-4}$	$p=8.881\times10^{-4}$	$p=0.048$	
lvs.m/root.m	g g$^{-\beta}$	log	1.293***	0.388**	0.090	0.354**	0.316
			$\pm$0.074	$\pm$0.105	$\pm$0.093	$\pm$0.111	
			$p=2.664\times10^{-17}$	$p=1.882\times10^{-3}$	$p=0.394$	$p=3.287\times10^{-3}$	
lvs.m/shoot.m	g g$^{-\beta}$	exp	2.124***	0.225**	-0.089	0.201*	0.391
			$\pm$0.049	$\pm$0.070	$\pm$0.062	$\pm$0.074	
			$p=1.499\times10^{-28}$	$p=5.502\times10^{-3}$	$p=0.201$	$p=0.011$	
n.trunks	count	log	0.655***	-0.013	-0.552**		0.254
			$\pm$0.147	$\pm$0.117	$\pm$0.175		
			$p=9.666\times10^{-5}$	$p=0.921$	$p=6.240\times10^{-3}$		
N_{area}	mg cm^{-2}	sqrt	0.447***	-0.050***	-0.034***		0.831
			$\pm6.183\times10^{-3}$	$\pm4.917\times10^{-3}$	$\pm7.390\times10^{-3}$		
			$p=3.952\times10^{-36}$	$p=3.140\times10^{-10}$	$p=2.329\times10^{-4}$		
N_{mass}	mg g^{-1}	log	2.918***	-0.061	0.193**		0.324
			$\pm$0.043	$\pm$0.034	$\pm$0.052		
			$p=3.437\times10^{-35}$	$p=0.112$	$p=1.729\times10^{-3}$		
$NDVI_{adax}$	unitless	exp	2.266***	0.026	0.168**		0.367
			$\pm$0.036	$\pm$0.029	$\pm$0.043		
			$p=3.898\times10^{-34}$	$p=0.421$	$p=1.270\times10^{-3}$		
pore.length	μm^2	log	2.415***	-0.012	-0.103*		0.239
			$\pm$0.030	$\pm$0.024	$\pm$0.035		
			$p=9.674\times10^{-38}$	$p=0.643$	$p=0.011$		
ψ_{md}	MPa	log	-0.430***	0.314***	0.066		0.56
			$\pm$0.067	$\pm$0.053	$\pm$0.080		
			$p=3.686\times10^{-7}$	$p=6.504\times10^{-6}$	$p=0.464$		
ψ_{pd}	MPa	log	0.484***	0.647***	0.051		0.605
			$\pm$0.122	$\pm$0.097	$\pm$0.146		
			$p=4.100\times10^{-4}$	$p=1.025\times10^{-6}$	$p=0.768$		
$refl_{adax}$	unitless	log	-2.836***	-0.052	-0.340**		0.292

continued

Trait	Units	Trans	Intercept	site P/E_p	species P/E_p	Interact	R^2
			±0.087	±0.069	±0.104		
			p =1.614×10^{-25}	p =0.511	p =4.897×10^{-3}		
$refl_{adax/abax}$	unitless	log	-0.311***	-0.053	−0.110°		0.203
			±0.045	±0.036	±0.054		
			p =1.056×10^{-7}	p =0.185	p =0.072		
$RGR.height$	cm cm^{-1} day^{-1}		2.632***	1.392***	0.973***	0.933***	0.828
			±0.108	±0.153	±0.137	±0.162	
			p =2.423×10^{-21}	p =6.299×10^{-9}	p =4.482×10^{-7}	p =2.747×10^{-6}	
$RGR.shoot$	g g^{-1} day^{-1}		6.009***	2.539***	2.206***	2.141***	0.771
			±0.230	±0.326	±0.291	±0.345	
			p =3.208×10^{-22}	p =1.180×10^{-7}	p =1.737×10^{-7}	p =7.765×10^{-7}	
$root.m/shoot.m$	g g$^{-\beta}$	sqrt	0.602***	-0.066*	-3.697×10^{-3}	-0.063*	0.203
			±0.018	±0.026	±0.023	±0.027	
			p =2.458×10^{-25}	p =0.023	p =0.889	p =0.027	
$shoot-lig.mass$	g	log	7.364***	1.185**	2.229***	0.883*	0.693
			±0.252	±0.358	±0.319	±0.378	
			p =1.274×10^{-23}	p =4.570×10^{-3}	p =5.451×10^{-7}	p =0.026	
$shoot.m/height$	g cm$^{-\beta}$	log	-5.371***	-0.254**	-0.128		0.327
			±0.094	±0.075	±0.113		
			p =6.070×10^{-33}	p =3.866×10^{-3}	p =0.305		
SLA	cm^2 g^{-1}	log	4.526***	0.138***	0.328***		0.777
			±0.035	±0.028	±0.042		
			p =5.988×10^{-44}	p =7.801×10^{-5}	p =8.569×10^{-8}		
$sPRI_{adax}$	unitless	exp	1.638***	0.011***	-0.012**		0.444
			±3.061×10^{-3}	±2.434×10^{-3}	±3.659×10^{-3}		
			p =4.819×10^{-63}	p =3.901×10^{-4}	p =4.439×10^{-3}		
$stomate.size$	μm^2	log	6.218***	0.069	−0.117°		0.147
			±0.051	±0.041	±0.061		
			p =4.979×10^{-43}	p =0.129	p =0.089		
$survival$	portion		0.905***	0.541***	0.017	0.345***	0.674
			±0.055	±0.078	±0.069	±0.082	
			p =1.152×10^{-16}	p =5.451×10^{-7}	p =0.839	p =2.141×10^{-4}	
TLP	MPa	log	-0.522***	0.112***	0.093**		0.625
			±0.025	±0.020	±0.030		
			p =7.144×10^{-20}	p =1.311×10^{-5}	p =6.335×10^{-3}		
$V_{cmax}/mass$	μmol g^{-1} s^{-1}	sqrt	0.959***	0.039	0.103*		0.272
			±0.032	±0.026	±0.039		
			p =2.621×10^{-24}	p =0.180	p =0.019		
VLA	μm mm^{-2}		8.382×10^3***	-1.337×10^3***	-1.903×10^3***		0.631
			±358.480	±285.120	±428.490		

continued

Trait	Units	Trans	Intercept	site P/E_p	species P/E_p	Interact	R^2
			p =2.973×10^{-21}	p =1.585×10^{-4}	p =2.958×10^{-4}		
WBI_{adax}	unitless		1.031***	-1.212×10^{-3}	-8.784×10^{-3}**		0.305
			±2.167×10^{-3}	±1.723×10^{-3}	±2.590×10^{-3}		
			p =1.859×10^{-61}	p =0.532	p =3.866×10^{-3}		
wood density	g cm^{-3}	sqrt	0.664***	-0.042***	-0.082***		0.711
			±0.011	±8.885×10^{-3}	±0.013		
			p =1.611×10^{-33}	p =1.272×10^{-4}	p =3.938×10^{-6}		
