# Supplementary material for: Ecophysiological adaptations shape distributions of closely related trees along a climatic moisture gradient
Source: Nat Commun. 2023 Nov 7;14:7173. doi: 10.1038/s41467-023-42352-w (PMC10630429; doi:10.1038/s41467-023-42352-w)
Supplement: Supplementary file 3 — Description of Additional Supplementary Files [file 41467_2023_42352_MOESM3_ESM.pdf]

Description of Additional Supplementary Files

File Name: Supplementary Data 1
Description: Summary of predicted patterns for each trait as a function of P/E_p.

File Name: Supplementary Data 2
Description: Summary of traits showing responses to species and site P/E_p which agree to predicted responses (see Supplementary Data 1 and Methods).
