# Supplementary material for: Ecophysiological adaptations shape distributions of closely related trees along a climatic moisture gradient
Source: Nat Commun. 2023 Nov 7;14:7173. doi: 10.1038/s41467-023-42352-w (PMC10630429; doi:10.1038/s41467-023-42352-w)
Supplement: Supplementary file 6 — Reporting Summary [file 41467_2023_42352_MOESM6_ESM.pdf]

nature portfolio

Corresponding author(s): Smith, Duncan D.
Givnish, Thomas J.

Last updated by author(s): Sep 13, 2023

Reporting Summary

Nature Portfolio wishes to improve the reproducibility of the work that we publish. This form provides structure for consistency and transparency in reporting. For further information on Nature Portfolio policies, see our Editorial Policies and the Editorial Policy Checklist.

Statistics

For all statistical analyses, confirm that the following items are present in the figure legend, table legend, main text, or Methods section.

n/a	Confirmed	
☐	☒	The exact sample size (*n*) for each experimental group/condition, given as a discrete number and unit of measurement
☐	☒	A statement on whether measurements were taken from distinct samples or whether the same sample was measured repeatedly
☐	☒	The statistical test(s) used AND whether they are one- or two-sided<br>*Only common tests should be described solely by name; describe more complex techniques in the Methods section.*
☐	☒	A description of all covariates tested
☐	☒	A description of any assumptions or corrections, such as tests of normality and adjustment for multiple comparisons
☐	☒	A full description of the statistical parameters including central tendency (e.g. means) or other basic estimates (e.g. regression coefficient) AND variation (e.g. standard deviation) or associated estimates of uncertainty (e.g. confidence intervals)
☐	☒	For null hypothesis testing, the test statistic (e.g. *F*, *t*, *r*) with confidence intervals, effect sizes, degrees of freedom and *P* value noted<br>*Give P values as exact values whenever suitable.*
☒	☐	For Bayesian analysis, information on the choice of priors and Markov chain Monte Carlo settings
☒	☐	For hierarchical and complex designs, identification of the appropriate level for tests and full reporting of outcomes
☒	☐	Estimates of effect sizes (e.g. Cohen's *d*, Pearson's *r*), indicating how they were calculated

Our web collection on statistics for biologists contains articles on many of the points above.

Software and code

Policy information about availability of computer code

Data collection	Data were collected using Open 6.3.4 (gas exchange), UniWin (spectral properties), Loggernet 4.7.0.15 (hydraulic conductance and turgor loss point), a bespoke data entry app in Pythonista
Data analysis	All analyses were performed using open-source resources including R 4.3.0 and packages within (phyr, rr2, stats) and ImageJ 1.53k

For manuscripts utilizing custom algorithms or software that are central to the research but not yet described in published literature, software must be made available to editors and reviewers. We strongly encourage code deposition in a community repository (e.g. GitHub). See the Nature Portfolio guidelines for submitting code & software for further information.

Data

Policy information about availability of data

All manuscripts must include a data availability statement. This statement should provide the following information, where applicable:

- Accession codes, unique identifiers, or web links for publicly available datasets
- A description of any restrictions on data availability
- For clinical datasets or third party data, please ensure that the statement adheres to our policy

The data that support the findings of this study are available from the corresponding author upon reasonable request

Human research participants

Policy information about studies involving human research participants and Sex and Gender in Research.

Reporting on sex and gender	N/A
Population characteristics	N/A
Recruitment	N/A
Ethics oversight	N/A

Note that full information on the approval of the study protocol must also be provided in the manuscript.

Field-specific reporting

Please select the one below that is the best fit for your research. If you are not sure, read the appropriate sections before making your selection.

☐ Life sciences ☐ Behavioural & social sciences ☒ Ecological, evolutionary & environmental sciences

For a reference copy of the document with all sections, see nature.com/documents/nr-reporting-summary-flat.pdf

Ecological, evolutionary & environmental sciences study design

All studies must disclose on these points even when the disclosure is negative.

Study description	Common garden study comparing many traits across species and environments. Factorial design (all species*garden combinations tested). Samples size typically 7 per combination. Larger sample sizes for survey measurements: height and survival. Analyses used environment (continuous) of site and species and their interaction as the predictive variables. Site or species were used as factors only when regressing PC1 against environment of species or site, respectively.
Research sample	Eucalyptus plants of ten species (E. arenacea, dives, dumosa, macrorhyncha, microcarpa, nitens, obliqua, regnans, sideroxylon, viminalis) planted at four common gardens in Victoria, Australia. Gardens span an environmental gradient of nominal water availability which the species also span in their native ranges. Plants were grown from seed and therefore are even aged and genetic makeup within species did not differ between sites.
Sampling strategy	We planted over 2000 individuals at each site to quantify survival well. We measured height of all living plants to quantify this well and determine the size distribution from which we chose focal plants. Typical samples sizes of 5-8 focal plants based of feasibility to perform all measurement in a short amount of time and typical sample sizes in the literature
Data collection	The first author collected most of the data, either storing it by the instrument performing the measurement or recording on paper or a bespoke data collection app.
Timing and spatial scale	Earliest data collected Nov 2017 and the latest Aug 2021. More detailed times shown in Fig S6. Most data came from three harvests intentionally spaced several months apart to be able to quantify processes including growth and survival. Within harvests, sites were sampled as close in time as logistics allowed so as to avoid differences due to seasonal changes.
Data exclusions	We removed impossible measurements including and associated with negative stomatal conductance. We removed leaf hydraulic conductance measures with low stem-to-leaf pressure difference (high noise to signal ratio). We removed measures of height, mass and growth for one individual, based on outlier tests. We removed and repeated measures of gmin for one site for methodological consistency. For PCA, we excluded some site*species combinations to produce a complete matrix. Exclusion criteria were not pre-established.
Reproducibility	We performed a preliminary study with fewer plants. In both preliminary and main studies we preformed many of the measurements at each harvest. When repeated, measurements could vary with ontogeny and season as expected but not changing conclusions
Randomization	Planting was randomized. Focal individuals were selected to span size range when size range was known. Otherwise selection was at random.
Blinding	Bias was avoided by choosing plants across their measured size range and humans performed no qualitative assessments. Many samples were labeled and assessed using a code rather than species name.

Did the study involve field work? ☒ Yes ☐ No

Field work, collection and transport

Field conditions	Fieldwork generally aimed to measure plants in sunny and dry conditions and was planned around lack of rain and moderate temperatures (for both plants and participants)
Location	Victoria Australia: , -36.72711 N 143.6085 E, -34.7389194 N 142.2502778 E, -37.39351 N 145.1415 E, -37.49107 N 145.5698 E. Elevations: 42, 280, 650 688 m.
Access & import/export	Common gardens were authorized by Department of Environment Land Water and Planning (permit NW11041F).
Disturbance	Common garden establishment required fencing areas, clearing vegetation and covering with weedmatting. Locations chosen in areas already mostly or fully clear. Sites will be rehabilitated at project end.

Reporting for specific materials, systems and methods

We require information from authors about some types of materials, experimental systems and methods used in many studies. Here, indicate whether each material, system or method listed is relevant to your study. If you are not sure if a list item applies to your research, read the appropriate section before selecting a response.

Materials & experimental systems

n/a	Involved in the study
☒	☐ Antibodies
☒	☐ Eukaryotic cell lines
☒	☐ Palaeontology and archaeology
☒	☐ Animals and other organisms
☒	☐ Clinical data
☒	☐ Dual use research of concern

Methods

n/a	Involved in the study
☒	☐ ChIP-seq
☒	☐ Flow cytometry
☒	☐ MRI-based neuroimaging
