# Supplementary material for: Ecophysiological adaptations shape distributions of closely related trees along a climatic moisture gradient
Source: Nat Commun. 2023 Nov 7;14:7173. doi: 10.1038/s41467-023-42352-w (PMC10630429; doi:10.1038/s41467-023-42352-w)
Supplement: Supplementary file 7 — Source Data [file 41467_2023_42352_MOESM7_ESM.zip › Source data file descriptions.pdf]

Source Data file descriptions for:

Ecophysiological adaptations shape distributions of closely related trees along a climatic moisture gradient

Duncan D. Smith[1,2,3], Mark A. Adams[2], Amanda M. Salvi[1], Christopher P. Krieg[1], Cécile Ané[1,4], Katherine A. McCulloh[1], Thomas J. Givnish[1]

[1]Department of Botany, University of Wisconsin-Madison, Madison, WI 53706, USA

[2]Faculty of Science, Engineering, & Technology, Swinburne University of Technology, Hawthorn, Victoria 3122, Australia

[3]School of Ecosystem and Forest Sciences, University of Melbourne, Creswick, Victoria 3363 Australia

[4]Department of Statistics, University of Wisconsin-Madison, Madison, WI 53706, USA

Corresponding authors: Duncan D. Smith (ddsmith3@wisc.edu)

Thomas J. Givnish (givnish@wisc.edu)

australia grid.csv

Center points of 0.25 degree pixels across a rectangular grid that includes Australia. Data fields are latitude, longitude, and boolean values indicating if the point is on land and if it is within the state of Victoria. This file is provided to create boundaries in Fig 1b.

euc site colors.csv

Plotting symbol and naming information for each of the four common garden sites. This file is only provided to add functionality to shared R scripts

euc species colors.csv

Plotting symbol and naming information for each of the ten study species (plus E. tricarpa). This file is only provided to add functionality to shared R scripts

euc tree.tre

A phylogeny of the ten study species (plus E. tricarpa) pruned from the larger phylogeny produced by Thornhill et al[46]. This file is provided to create Fig 1b and to perform phylogenetically structure regressions.

Fig 1bcd.csv

Occurrence and P/Ep for the 10 study species within 0.25 degree pixels. These data were extracted from the Atlas of Living Australia (occurrence), WorldClim (P) and the Bureau of Meteorology (Ep). This file provides data to create the elements of Fig 1b,c,d and is is the basis for species' mean P/Ep values used in the study.

Fig 2,3,4,S2,S3,S4.csv

Trait means (transformed as needed) for all trait*species*site combinations included in the PCA. This long-format of the data also includes units and descriptions of each trait. Thile file provides the data for the PCA and all trait vs P/Ep regressions

Fig 5,6,S5,S6.csv

Means of height, mass, survival, height*survival and mass*survival as presented in the Figures noted in the file name.

Fig S1.csv

Historical monthly climate for the four common garden sites. Data were extracted from Worldclim and were used to create Walter and Leith diagrams (Fig S1).

Fig S11.csv

All records of mean survival for each site*species combinations. These data provide the basis for Fig S11.

Fig S7.csv

Soil volumetric water content during the study period at each of the four common gardens. These data provide the basis for Fig S7.

Fig S8.csv

Relative height growth at common garden Bealiba at three points in time. These data provide the basis for Fig S8.

Fig S9,S10.csv

Observed and historic monthly and annual temperature and rainfall for the four common garden sites. Historic data were extracted from Worldclim. These data provide the basis for Fig S9 and S10.

PCA data.csv

Trait means (transformed as needed) for all trait*species*site combinations included in the PCA. Values are the same as in "Fig 2,3,4,S2,S3,S4.csv" but in a format readily used to perform PCA in R.

trait means.csv

Trait means (transformed and untransformed) for all trait*species*site combinations included in the PCA and used for trait vs P/Ep regressions. transformed values are the same as in "Fig 2,3,4,S2,S3,S4.csv" and "PCA data.csv". This file is provided because untransformed means are needed to show traits in trait vs P/Ep plots.
